# Strong preference for autaptic self-connectivity of neocortical PV interneurons facilitates their tuning to γ-oscillations

**DOI:** 10.1371/journal.pbio.3000419

**Published:** 2019-09-04

**Authors:** Charlotte Deleuze, Gary S. Bhumbra, Antonio Pazienti, Joana Lourenço, Caroline Mailhes, Andrea Aguirre, Marco Beato, Alberto Bacci

**Affiliations:** 1 ICM–Institut du Cerveau et de la Moelle épinière, Inserm U1127, CNRS UMR 7225, Sorbonne Université, Paris, France; 2 Department of Neuroscience, Physiology and Pharmacology, University College London, London, United Kingdom; 3 European Brain Research Institute, Rome, Italy; Institute of Science and Technology Austria, AUSTRIA

## Abstract

Parvalbumin (PV)-positive interneurons modulate cortical activity through highly specialized connectivity patterns onto excitatory pyramidal neurons (PNs) and other inhibitory cells. PV cells are autoconnected through powerful autapses, but the contribution of this form of fast disinhibition to cortical function is unknown. We found that autaptic transmission represents the most powerful inhibitory input of PV cells in neocortical layer V. Autaptic strength was greater than synaptic strength onto PNs as a result of a larger quantal size, whereas autaptic and heterosynaptic PV-PV synapses differed in the number of release sites. Overall, single-axon autaptic transmission contributed to approximately 40% of the global inhibition (mostly perisomatic) that PV interneurons received. The strength of autaptic transmission modulated the coupling of PV-cell firing with optogenetically induced γ-oscillations, preventing high-frequency bursts of spikes. Autaptic self-inhibition represents an exceptionally large and fast disinhibitory mechanism, favoring synchronization of PV-cell firing during cognitive-relevant cortical network activity.

## Abstract 2

Parvalbumin-positive interneurons modulate cortical activity via highly specialized connections to excitatory pyramidal neurons and other inhibitory cells. However, this study shows that fast autaptic self-inhibition is the major output of parvalbumin-positive basket cells in the neocortex and serves to modulate phase-locking of these interneurons during gamma-oscillations.

## Introduction

In the neocortex, cognitive-relevant processes depend on the activity of intricate networks formed by specific excitatory and inhibitory neuronal populations that are interconnected according to a detailed blueprint [1–4]. In particular, fast synaptic inhibition governs both spontaneous and sensory-evoked cortical activity and originates from a rich diversity of cell types with precisely distinct functions within cortical circuits [4,5]. Perisomatic-targeting parvalbumin (PV)-expressing basket cells represent a major population of cortical GABAergic neurons. By providing fast inhibition onto pyramidal neuron (PN) cell bodies, PV cells exert a fine control of their output gain [4,6] and spike timing, resulting in the generation and modulation of γ-rhythms, important for sensory perception and attention [7–10]. Indeed, in awake animals, PV cells fire trains of spikes, which are strongly phase locked to both spontaneous and visually evoked γ-rhythmic activity [11].

In addition to targeting PNs, PV cells strongly inhibit one another, and GABAergic connections between PV cells are the major source of inhibition for this interneuron type [12,13]. Moreover, PV cells are self-connected by autapses (synapses that a neuron makes with itself [14]). Self-inhibition was first described anatomically in adult neocortex of the cat [15], and it was demonstrated to be functional in rodent [16–20] and human neocortex [21,22]. In particular, fast autaptic neurotransmission plays a crucial role in setting millisecond-precise spike timing of PV cells during trains of action potentials (APs) [17]. Moreover, high-frequency firing of PV cells triggers massive asynchronous autaptic release of GABA, resulting in prolonged PV-cell self-inhibition that desynchronizes PV-cell firing [20–22].

Connections between PV cells form a specific inhibitory network that is important for synchronizing a large population of neurons during γ-oscillations [7,8,23]. Despite the known role of PV cells as the clockwork of cortical networks, the underlying mechanism is still poorly understood. In addition, although functional autaptic transmission was demonstrated, the actual proportion of self-connections in relation to other synaptic projections from neocortical PV cells to other neurons is unknown. Are autapses solely a connectivity curiosity, or do they represent an important source of inhibition of PV cells? Could fast self-inhibition contribute in keeping PV-cell firing in sync with rhythmic network activity?

Here, we measured the strength of autaptic self-inhibition compared with synaptic transmission from the same PV cell onto their two principal synaptic targets: PNs and other PV cells. Remarkably, autaptic responses were invariably much larger than unitary synaptic transmission onto PNs and, on average, onto other PV cells. Quantal synaptic parameters underlying the autaptic versus synaptic strength were different depending on whether the postsynaptic neuron was a PN or another PV cell. We found that PV cells with strong autaptic inhibition provided little input to other PV cells, whereas PV cells with smaller autapses provided larger heterosynaptic inhibition to neighboring PV cells. Remarkably, self-connections accounted for up to approximately 40% of the perisomatic inhibitory strength onto single PV cells. Finally, we found that autaptic transmission tuned the strong coupling of PV-cell spikes with γ-oscillations by modulating spike after-hyperpolarization (AHP) and, thus, interspike intervals (ISIs). Therefore, autaptic self-innervation accounts for a large fraction of the synaptic inhibition PV cells receive and seems to improve the tuning of their spiking activity with cognitive-relevant network oscillations.

## Results

### Layer V PV cells connect more powerfully with themselves via autaptic contacts than with other synaptic partners

**Fig 1:**
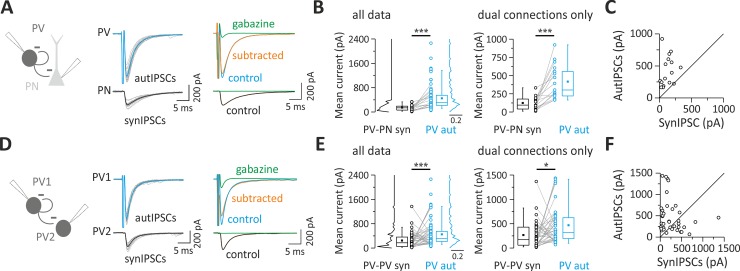
Layer V PV cells connect more powerfully with themselves via autaptic contacts than with other synaptic partners. (A) Unitary autaptic and synaptic inhibitory currents (autIPSCs and synIPSCs) evoked simultaneously in a PV cell and a PN, respectively, in response to PV cell stimulation. Individual responses (15 gray traces) were averaged (thick trace, blue for autIPSC and black for synIPSC). In the presence of the GABA_A_R antagonist, gabazine (10 μM), the two responses were blocked, but note the residual current in the PV cell reflecting the distortion due to the voltage step eliciting the action potential current (clipped). In order to cancel this stimulus waveform, current traces in gabazine were subtracted from control responses (orange: subtracted trace, average of 10 trials). (B) Population data obtained from PV-PN pairs with either single (autaptic or synaptic) or paired dual (autaptic and synaptic) connections (all data, left panel). Right panel illustrates pairs with both synaptic and autaptic connections from the same presynaptic PV cell (dual connections only). Note that the mean autaptic current from PV cell is systematically and significantly larger than the synaptic one (***p < 0.001). (C) Representative traces of autIPSCs and synIPSCs as in (A) but recorded in a PV-PV pair. (D) Population data obtained from PV-PV pairs with summary plots as described in (B). Note that, on average, autaptic currents are larger than synaptic currents (***p < 0.001, *p < 0.05). Individual numerical data for panels B, C, E, and F are provided in Supporting information, S1 Data. autIPSC, autaptic inhibitory postsynaptic current; GABA_A_R, GABA_A_ receptor; PN, pyramidal neuron; PV, parvalbumin; synIPSC, synaptic inhibitory postsynaptic current.

In order to compare autaptic inhibition of PV cells with the synaptic inhibition from the same PV cells through GABAergic connections onto PNs and other PV cells, we performed simultaneous paired recordings between these two cell types in neocortical layer V of the mouse somatosensory (barrel) cortex of acute brain slices. We used a transgenic mouse strain to identify PV cells unambiguously (PV-cre::TdTomato; see Materials and methods). Briefly, tdTomato-positive neurons exhibit a clear multipolar morphology and stereotypical fast-spiking behavior. Nonfluorescent PNs had typical large cell bodies and an apical dendrite directed toward the pia (see Materials and methods). We isolated GABAergic events pharmacologically and used a high-Cl intracellular solution (see Materials and methods) for voltage-clamp recordings of GABAergic synaptic currents that were inward at a holding potential of −80 mV. We elicited action currents in PV cells in voltage clamp by delivering brief (0.2–0.6 ms) depolarizing steps from −80 mV to membrane potential between −20 mV and 0 mV in order to minimize passive electrical artifacts induced by the stimulus. Self-connected PV cells exhibited large GABAergic inward responses following action currents. As previously demonstrated [16,17], these responses result from unitary autaptic transmission because they exhibit fixed latencies and peak amplitude fluctuations and were abolished by the GABA_A_ receptor (GABA_A_R) antagonist gabazine (10 μM, Fig 1A). We found GABAergic autaptic inhibitory postsynaptic currents (autIPSCs) in 74% of recorded PV neurons (*n* = 164). The same action currents in PV cells elicited unitary synaptic inhibitory postsynaptic currents (synIPSCs) onto a fraction of PNs (Fig 1A and 1B) or PV cells (Fig 1C and 1D). Both aut- and synIPSCs had fast decay time constants (<10 ms), and autaptic transmission was on average faster than unitary synaptic transmission onto PNs (*p* = 1.87 × 10^−6^; Mann–Whitney–Wilcoxon test; Table 1).

**Table 1:** Mean current and kinetics in all PV-PN and PV-PV pairs.

All data	Amplitude (pA)	Decay time constant (ms)
PV autaptic IPSCs	451.82 ± 43.14; *n* = 84	3.69 ± 0.16; *n* = 14
PV-PN synaptic IPSCs	146.87 ± 20.89; *n* = 22	7.30 ± 0.30; *n* = 18
PV-PV synaptic IPSCs	246.11 ± 36.15; *n* = 49	4.13 ± 0.28; *n* = 20

Abbreviations: IPSC, inhibitory postsynaptic current; PN, pyramidal neuron; PV, parvalbumin

The yield of finding connected PV-PN pairs was of 61% (36 out of 59 pairs), of which 75% also exhibited autaptic responses (27 out of 36 pairs). We found that PV-PN responses were invariably much smaller than their autaptic counterparts, either when they were analyzed independently (Table 1; *p* = 5.6 × 10^−7^, *n* = 84 and 22 for autaptic and synaptic transmission, respectively) or when paired dual connections were analyzed separately (Table 2; *p* = 5.3 × 10^−4^, *n* = 16; Fig 1B).

**Table 2:** Mean current in PV-PN and PV-PV pairs with both autaptic and synaptic connections.

	Dual connections only	
Recorded pairs	Autaptic IPSCs (pA)	Synaptic IPSCs (pA)
PV-PN (*n* = 16)	417.50 ± 58.10	124.32 ± 21.92
PV-PV (*n* = 38)	469.99 ± 62.48	266.59 ± 44.44

Abbreviations: IPSC, inhibitory postsynaptic current; PN, pyramidal neuron; PV, parvalbumin

Among pairs between PV cells, the proportion of connected synaptic PV-PV pairs was 61% (59 out of 96 pairs), of which 76% also exhibited autaptic responses (45 out of 59 pairs). Also, in this case, PV-cell autaptic strength was larger than PV-PV synaptic transmission (Table 1; *p* = 5.7 × 10^−5^, *n* = 84 and 49 for autaptic and synaptic transmission, respectively), both when autIPSCs and synIPSCs were analyzed independently and when paired dual connections originating from the same PV cell were analyzed separately (Table 2; *p* = 0.0215, *n* = 38; Fig 1D). PV-PV unitary synaptic and autaptic responses had similar decay time constant (*p* = 0.2; Table 1).

These results indicate that autaptic self-inhibition of PV cells is more powerful than synaptic transmission from the same PV cells onto their principal postsynaptic targets in layer V: PNs and other PV cells.

### Quantal parameters accounting for larger unitary autaptic than synaptic connections between PV cells and PNs

Synaptic efficacy results from the combination of pre- and postsynaptic factors—namely, the number of presynaptic release sites (n), the probability of neurotransmitter release (p), and the postsynaptic response to a single released synaptic vesicle (or quantum), known as quantal size (q). This can be summarized by expressing the average unitary synaptic current <I_syn_> as a product of the quantal parameters:

$$<I_{syn}>=npq$$

**Fig 2:**
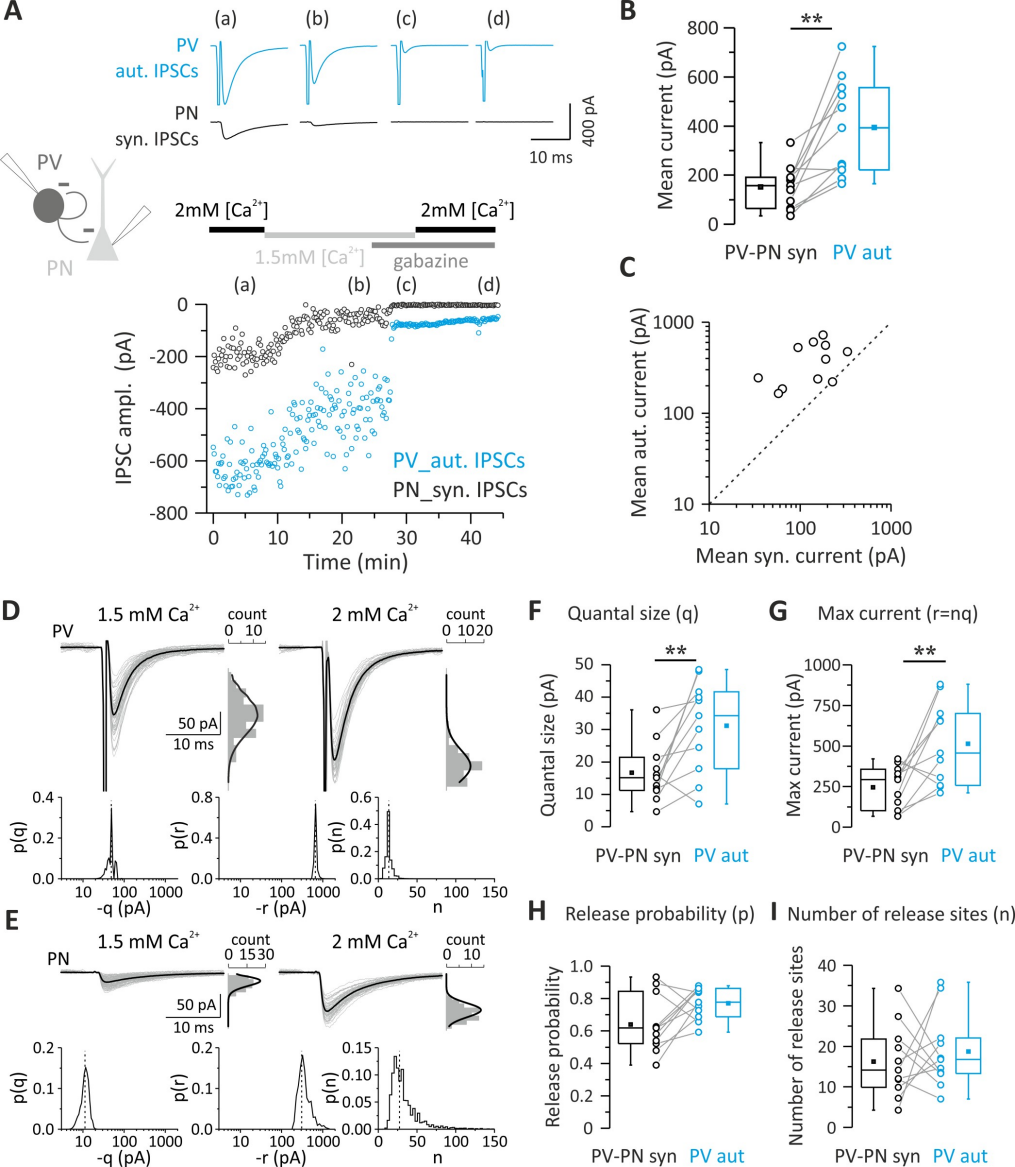
Quantal parameters accounting for larger unitary autaptic than synaptic connections between PV cells and PNs. (A) Top: representative autaptic and synaptic traces in response to PV-cell stimulation recorded from a PV-PN pair. IPSCs were elicited every 10 s, and each trace is the average of 10 sweeps. Bottom: time course of autIPSC (blue symbols) and synIPSC (black symbols) amplitude recorded simultaneously from the same PV cell and PN, respectively, at two extracellular Ca^2+^ concentrations (2.0 and 1.5 mM) and in the presence of 10 μM gabazine (at both [Ca^2+^]) to subtract the stimulus waveform. (B, C) Summary (B) and correlation plots (C) of synIPSCs and autIPSC amplitude obtained from all PV-PN pairs with dual connections used for BQA and measured at 2 mM [Ca^2+^]. Note that in all pairs (open circles), autaptic currents in PV cells are consistently bigger than their synaptic correlates in PN, as all points fall above the unity line (dashed line). (D–I) Quantal analysis of PV and PN responses to PV-cell stimulation recorded from the PV-PN pair shown in (A). Example responses of the PV cell (D) and PN (E) are shown alongside their respective amplitude distributions observed in the presence of 1.5 mM (left, low release probability) and 2 mM (right, high release probability) extracellular Ca^2+^. The results of BQA are represented as probability distributions for the quantal size (q) (F), maximal response (n) (G), release probability (p) (H), and number of release sites (r) (I). The dashed line is the median. Note the larger quantal size and maximum current of autaptic responses (*n* = 11; **p < 0.01). Individual numerical data for panels B, C, F, G, H, and I are provided in Supporting information, S2 Data. ampl., amplitude; autIPSC, autaptic inhibitory postsynaptic current; BQA, Bayesian quantal analysis; IPSC, inhibitory postsynaptic current; PN, pyramidal neuron; PV, parvalbumin; synIPSC, synaptic inhibitory postsynaptic current.

Autaptic self-inhibition onto PV cells is powerful and, on average, stronger than synaptic transmission from the same cells to other elements of cortical microcircuit (Fig 1). We therefore set out to determine the quantal parameter(s) responsible for stronger autaptic neurotransmission using Bayesian quantal analysis (BQA) [24]. We recorded from pairs of PV cells and PNs exhibiting GABAergic autaptic and synaptic responses at two extracellular Ca^2+^ concentrations (2.0 and 1.5 mM), resulting in different release probabilities. At the end of each recording, we applied the GABA_A_R antagonist gabazine to enable subtraction of the stimulus waveform and action current to isolate autaptic responses for each of the Ca^2+^ concentrations (Fig 2A). Also, in this set of data, autIPSCs recorded with high [Ca^2+^] (2 mM) were invariably larger than synIPSCS (mean current = 394.15 ± 58.54 versus 151.64 ± 26.24 pA; autaptic versus synaptic connections; *p* = 0.002, *n* = 11; Fig 2B and 2C). We then applied BQA at unitary autaptic and synaptic responses recorded at low ([Ca^2+^] = 1.5 mM) and high ([Ca^2+^] = 2 mM) release probabilities and obtained median-based estimates for the quantal parameters from the marginal posterior distributions for the quantal size q and maximal response r (where r = nq) and, hence, number of release sites n (Fig 2D and 2E) [24].

We found that in PV-PN pairs with both autaptic and synaptic connections, autaptic responses had a significantly larger quantal size (q) than unitary synaptic connections onto PNs (Table 3; *p* = 0.00976, *n* = 11; Fig 2F). Accordingly, the maximal response r (nq) was also larger in autaptic versus synaptic responses onto PNs (Table 3; *p* = 0.0098, *n* = 11; Fig 2G). Conversely, no differences in release probability (p) and number of release sites (n) were shown by comparison of autaptic transmission onto PV cells and synaptic inhibition from the same neurons onto PNs (Table 3; *p* > 0.05 *n* = 11; Fig 2H and 2I).

**Table 3:** Bayesian quantal analysis in PV-PN pairs (*n* = 11) with both autaptic and synaptic connections.

Quantal parameter	Autaptic IPSCs	Synaptic IPSCs
Quantal size (q)	31.17 ± 4.28 pA	16.68 ± 2.71 pA
Number of release site (n)	18.70 ± 2.76	16.26 ± 2.70
Probability of release (p)	0.77 ± 0.03	0.64 ± 0.05
Maximum current (r)	513.65 ± 74.82 pA	245.33 ± 39.88 pA

Abbreviations: IPSC, inhibitory postsynaptic current; PN, pyramidal neuron; PV, parvalbumin

To test whether our BQA approach yielded a valid estimate of q, we assessed quantal size using an independent approach. We have previously demonstrated that PV cells can generate strong asynchronous GABA release in response to high-frequency AP trains both at autaptic sites and at synapses with PNs and other PV cells [20], allowing an estimate of the quantal size of unitary connections. We performed paired PV-PN recordings and induced autaptic and synaptic asynchronous release in response to high-frequency trains (200 Hz; 1–2 s). We then generated amplitude distributions of events detected in a time window of 400–500 ms immediately after the trains. We found that median amplitude values of asynchronous autaptic events were significantly larger than their synaptic counterparts in PNs (S1 Fig). Moreover, quantal size estimated from autaptic and synaptic asynchronous events was very similar to that obtained with BQA (mean asynchronous IPSC amplitudes: 36.67 ± 4.37 versus 18.02 ± 2.27 pA; *n* = 10 and 7 for autaptic and synaptic events, respectively; mean quantal size [BQA]: 31.17 ± 4.28 versus 16.68 ± 2.71 pA; *n* = 11 for autaptic versus synaptic transmission onto PNs, respectively).

These results indicate a larger quantal size at autaptic sites as compared with synapses that the same PV cells formed with PNs. This is consistent with PV-PN synaptic transmission being invariably smaller than autaptic transmission. Therefore, stronger autaptic efficacy is likely due to cell type–specific postsynaptic mechanisms.

### The strength of autaptic and synaptic transmission onto PV cells depends on different number of release sites

**Fig 3:**
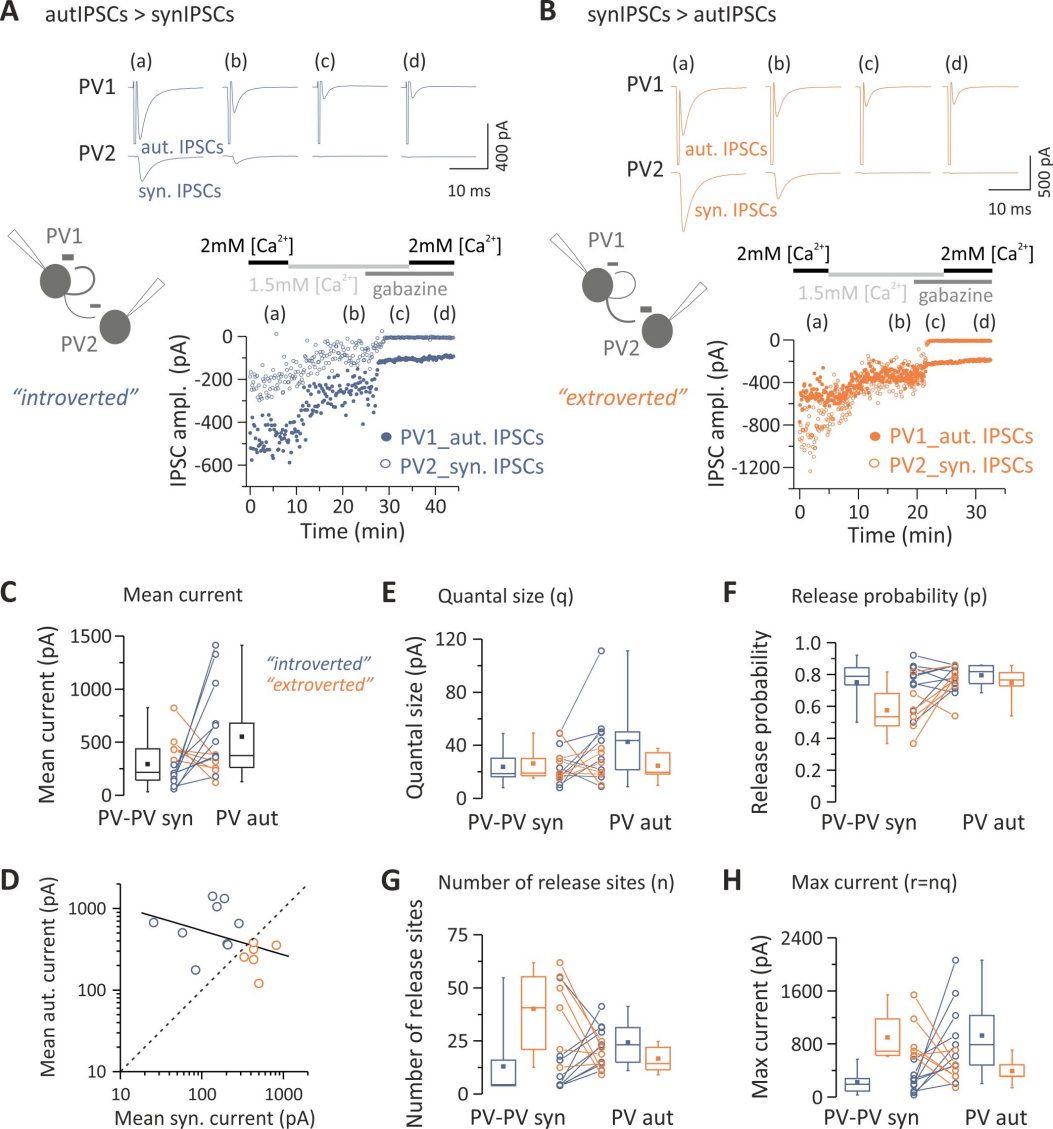
Extroverted and introverted PV cells rely on a different number of release sites. (A, B) Top: representative responses from pairs of PV cells (PV1 and PV2) at two extracellular Ca^2+^ concentrations (2.0 and 1.5 mM) and in the presence of gabazine. Shown are cases of introverted (autIPSCs > synIPSCs [A]) and extroverted (autIPSCs < synIPSCs [B]) presynaptic PV cells. Each trace is the average of 10 sweeps. Bottom: time course of autaptic and synaptic IPSC amplitudes. Responses were elicited every 10 (A) or 5 s (B). (C) Summary plots of synIPSC and autIPSC amplitude from all pairs used for BQA (black box charts), measured at 2 mM [Ca^2+^], with color-coded introverted and extroverted PV cells (blue and orange, respectively). (D) autIPSCs plotted against synIPSCs. Data were fitted with a linear regression (black line) showing a good degree of correlation between paired autaptic and synaptic responses from a single presynaptic PV cell. Note also that individual pairs (open circles) are distributed on both sides of the relationship for equal autaptic and synaptic IPSCs (dashed line), thus indicating a split of PV cells into two types of connection patterns, introverted and extroverted (blue and orange, respectively). (E–H) Quantal analysis of PV responses. Introverted and extroverted cases are color coded as in (A). Results of BQA are represented as probability distributions for the quantal size (q) (E), release probability (p) (F), number of release sites (n) (G), and max response (r) (H). Note that the large size of an autaptic/synaptic response relies on a large number of release sites compared with their synaptic/autaptic correlate in all but one introverted and extroverted PV cell, respectively. Individual numerical data for panels C–H are provided in Supporting information, S3 Data. ampl., amplitude; autIPSC, autaptic inhibitory postsynaptic current; BQA, Bayesian quantal analysis; IPSC, inhibitory postsynaptic current; max, maximal; PV, parvalbumin; synIPSC, synaptic inhibitory postsynaptic current.

Pairs of PV cells were analyzed to determine whether differences in unitary autaptic versus synaptic transmission onto other PV cells could be accounted for by any of the quantal parameters. We noticed that the strength of self- versus heterosynaptic inhibition defined two connectivity patterns of PV cells: those with stronger autaptic than synaptic PV-PV connections and those that showed an opposite trend (referred to as “introverted” and “extroverted” PV cells, respectively; Fig 3A and 3B). In our hands, introverted PV cells (in which autIPSCs > synIPSCs) corresponded to 63.1% of the total dual connected sample (*n* = 24 out of 38). Of those 38 PV cells, stable experiments suitable for BQA analysis were obtained in 15 pairs, nine of which were introverted, and the remaining six were extroverted PV cells (corresponding to 60% and 40%, respectively; Fig 3C and 3D). Importantly, the size of autaptic and unitary synaptic response was inversely correlated (R = −0.5643, *p* = 0.031; Fig 3D), suggesting the existence of two connectivity patterns between PV cells that could be distinguished by their self-inhibition strength. We found that in both introverted and extroverted PV cells, autaptic and synaptic quantal size (q) was similar for both introverted and extroverted PV cells (*n* = 9 and 6, respectively; Fig 3E). In addition, release probability (p) was similar for self- and PV-PV synaptic inhibitory contacts (Fig 3F). However, we found that a differential number of release sites (n) determined the strength of autaptic and synaptic connections onto PV cells. Indeed, in eight out of nine introverted PV-cell pairs, the number of release sites (n) was larger in autaptic than synaptic connections (Table 4; Fig 3G, orange symbols). Accordingly, the opposite was true for extroverted PV-cell pairs, in which in five out of six cases, the number of autaptic release sites was smaller than that of PV-PV synaptic connections (Table 4; Fig 3G, blue symbols). Therefore, the maximal autaptic response r (or nq) was larger or smaller in introverted and extroverted PV-cell pairs, respectively (Table 4; Fig 3H).

**Table 4:** Bayesian quantal analysis in PV-PV pairs with both autaptic and synaptic connections.

	autIPSCs > synIPSCs (*n* = 9)		autIPSCs < synIPSCs (*n* = 6)	
Quantal parameter	autIPSCs	synIPSCs	autIPSCs	synIPSCs
Quantal size (q)	42.54 ± 10.10 pA	23.82 ± 4.60 pA	24.47 ± 4.32 pA	26.36 ± 5.18 pA
Number of release site (n)	24.34 ± 3.29	13.03 ± 5.52	16.75 ± 2.51	40.18 ± 8.02
Probability of release (p)	0.80 ± 0.02	0.75 ± 0.05	0.75 ± 0.05	0.58 ± 0.06
Maximum current (r)	927.72 ± 198.28 pA	222.01 ± 54.94 pA	393.30 ± 78.53 pA	900.61 ± 153.58 pA

Abbreviations: autIPSC, autaptic inhibitory postsynaptic current; PV, parvalbumin; synIPSC, synaptic inhibitory postsynaptic current

These results indicate that the strength of autaptic transmission in PV cells, as compared with heterosynaptic PV-PV connections, is determined by the number of release sites and, thus, can be accounted for by structural differences, in contrast with PV-PN connections, in which the greater strength of autaptic versus heterosynaptic currents is mainly determined by differences in the quantal size.

### Autaptic neurotransmission accounts for a large fraction of the total inhibition onto single PV cells

**Fig 4:**
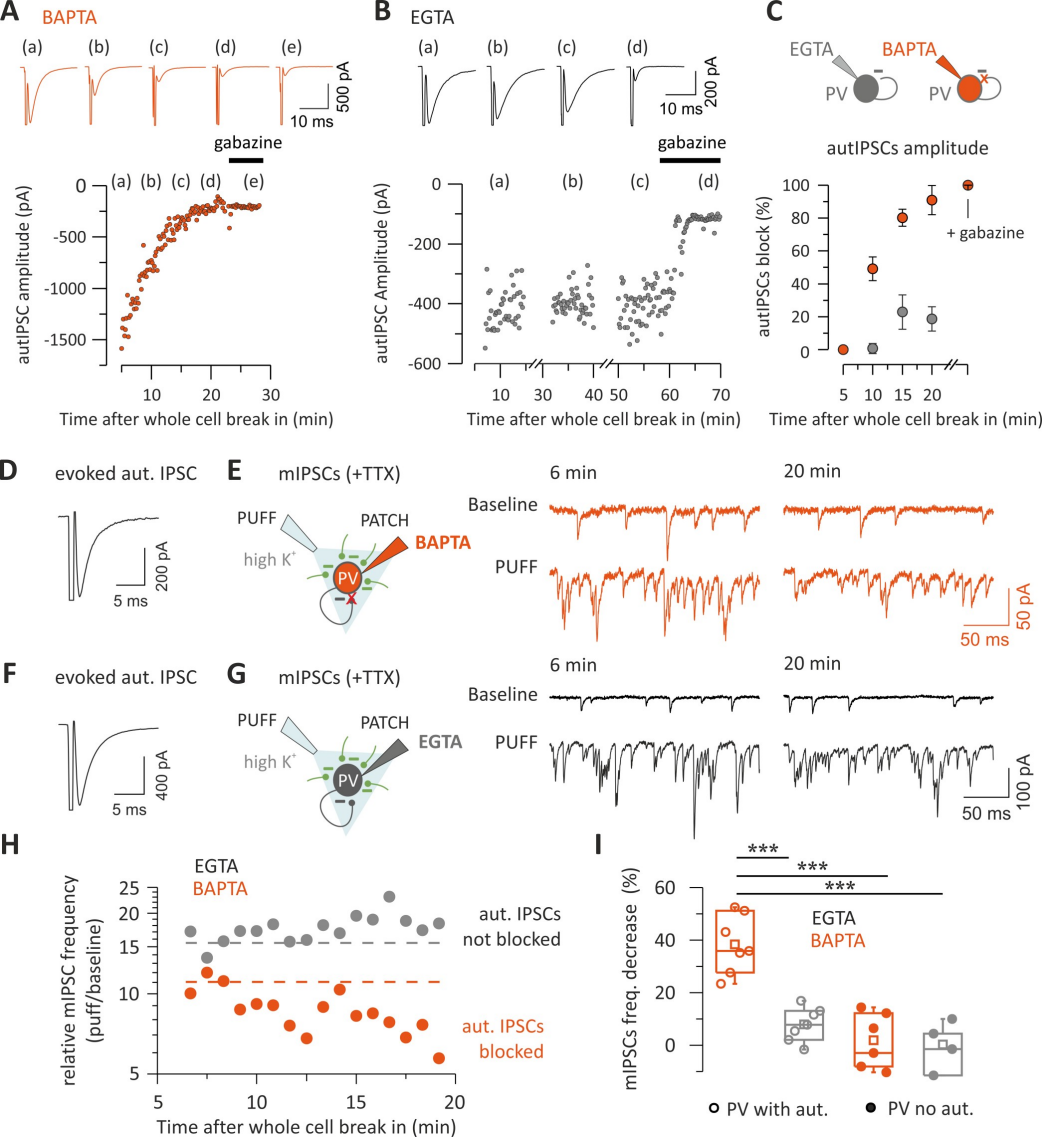
Autaptic neurotransmission accounts for a large fraction of the total inhibition onto single PV cells. (A–C) Representative voltage-clamp traces (top) and time course (bottom) of autaptic responses recorded from two PV cells in the presence of 20 mM BAPTA (A, orange) or 1 mM EGTA (B, black) in the recording whole-cell pipette. IPSCs were elicited every 10 s, and their amplitudes illustrated on the time course plot (bottom). Note the decline of autIPSC amplitudes during BAPTA perfusion up to a complete block (A), as compared with the absence of rundown during long (1 h) EGTA perfusion (B). Gabazine completely blocked autIPSCs in both cases. (C) Summary of autaptic IPSC block by intracellular BAPTA in PV cells. IPSC amplitudes were normalized to the average value obtained 5 min after establishment of whole-cell configuration. Autaptic currents are blocked by BAPTA perfusion within 20 min (*n* = 5) but not in EGTA (*n* = 5). (D) Average trace of autIPSC recorded in a PV cell immediately after whole-cell establishment in the presence of BAPTA in the recording pipette. (E) Schematic of the experiment (left) and representative voltage-clamp traces of mIPSCs before and after puffing high-K^+^ ACSF (PUFF) at the beginning (6 min) and after intracellular blockade of autaptic release (20 min). Note the decrease of mIPSC frequency in high extracellular K^+^ after 20 min of intracellular perfusion of BAPTA. (F, G) Same as in (E) and (F) but in another PV cell recorded with a control EGTA (1 mM) intracellular solution. Note that the increase of mIPSCs induced by high K^+^ is constant over the same period. (H) mIPSCs frequency changes induced by high-K^+^ puff in the same PV cells as in (E) and (G), as estimated by the ratio before and after application of the high-K^+^ solution for each puff. The dashed line indicates baseline frequency (average of the first three points). Note that in BAPTA, the frequency declines progressively following the same time course as the autaptic transmission block, whereas it is stable in EGTA. (I) The percentage of decrease in mIPSC frequency calculated between the same time points as in (E) and (G)—i.e., before and after potential autaptic transmission block—is shown on the summary plot. In PV cells with evoked autaptic IPSC, the frequency strongly decreases in the presence of intracellular BAPTA (*n* = 7) but not EGTA (*n* = 7), whereas in PV cells with no autaptic transmission, the frequency did not change in both conditions (BAPTA, *n* = 6; EGTA, *n* = 4) (***p < 0.001). Individual numerical data for panels C and I are provided in Supporting information, S4 Data. ACSF, artificial cerebrospinal fluid; autIPSC, autaptic inhibitory postsynaptic current; BAPTA, 1,2-bis(o-aminophenoxy)ethane-N,N,N′,N′-tetraacetic acid; IPSC, inhibitory postsynaptic current; mIPSC, miniature IPSC; PN, pyramidal neuron; PV, parvalbumin; TTX, tetrodotoxin.

The prevalence of self- versus synaptic GABAergic transmission originating from PV cells prompted the question of whether autaptic transmission provides a large proportion of the total synaptic inhibition that these cells receive. To measure the autaptic fraction contributing to the overall perisomatic inhibition received by single PV cells, we progressively blocked autaptic neurotransmission while evoking GABA release from virtually all terminals impinging the cell bodies of recorded PV cells. Autaptic transmission was blocked by intracellular perfusion of the membrane-impermeable free acid form of the fast Ca^2+^ chelator 1,2-bis(o-aminophenoxy)ethane-N,N,N′,N′-tetraacetic acid (BAPTA, 20 mM) in the presence of 2 mM Ca^2+^. Diffusion of BAPTA to autaptic contacts progressively reduced the size of autIPSCs until a complete block of autaptic neurotransmission was typically achieved within 20 min following whole-cell break in (Fig 4A). In order to rule out that this time-dependent reduction of autaptic responses was due to nonspecific rundown, we performed some control experiments, in which autIPSCs were recorded with an intracellular solution containing low concentration (1 mM) of the slow Ca^2+^ chelator EGTA, mimicking endogenous Ca^2+^ buffering by PV [25]. In the presence of intracellular EGTA, autIPSCs were stable for long periods (up to 1 h, Fig 4B) [16,20].

On average, after 20 min of intracellular BAPTA diffusion, autIPSC amplitude was 9.1% ± 8.8% of control (*n* = 5), whereas in the same timeframe, intracellular EGTA diffusion did not affect autaptic transmission (81.3% ± 7.4% of control; *n* = 5). To measure the relative fraction of autaptic inhibition onto PV cells, we first tested whether the recorded PV cell exhibited an autaptic response (Fig 4D); we subsequently applied the Na^+^ channel blocker tetrodotoxin (TTX, 1 μM) and measured a baseline period of miniature inhibitory postsynaptic currents (mIPSCs). Using a local micropipette, we then puffed artificial cerebrospinal fluid (ACSF) with a high concentration of KCl (20 mM) to depolarize all synaptic terminals impinging upon the recorded PV cell, thus forcing global Ca^2+^-dependent release of GABA without inducing unwanted network effects (Fig 4E; S2 Fig). We repeated the high-K^+^ puffs once per minute, for at least 20 min, and we measured the relative increase of mIPSC frequency (puff/baseline) as an estimate of global perisomatic inhibition onto the recorded cell (Fig 4D–4G).

In the presence of 20 mM intracellular BAPTA, the high-K^+^-dependent increase in mIPSC frequency declined steadily within 20 min after whole-cell break in Fig 4H, consistent with a complete autaptic blockade (Fig 4B). In contrast, in control experiments in which EGTA was internally diffused, the increase of mIPSC frequency was stable over the same period of time (Fig 4H). On average, mIPSC frequency blockade was 38.4% ± 4.2% and 7.9% ± 2.4% in the presence of BAPTA and EGTA, respectively (BAPTA *n* = 7; EGTA *n* = 7; *p* < 3.9 × 10^−5^; one-way ANOVA, followed by Tukey’s comparison; Fig 4I). Importantly, in those PV cells lacking autaptic responses, the high-K^+^-dependent increase of mIPSC frequency was stable over time, regardless of whether intracellular BAPTA or EGTA was present, ruling out nonspecific effects of BAPTA on mIPSCs (1.9% ± 4.3% and 0.3% ± 4.6%; *n* = 6 and 4, BAPTA and EGTA, respectively; *p* = 0.99, one-way ANOVA; Fig 4I).

We then used a different approach to confirm the approximately 40% estimate of the autaptic contribution to global synaptic inhibition that each PV cell receives. We measured single-cell (unitary) autaptic responses from PV cells and subsequently stimulated monosynaptic inhibitory afferents with an extracellular stimulating electrode in the continuous presence of glutamate receptor antagonist 6,7-dinitroquinoxaline-2,3,dione (DNQX). We set the stimulation intensity to obtain a quasi-maximal synaptic response, estimated by an input–output stimulation paradigm (see Materials and methods). We found that the ratio between autIPSC and evoked IPSC amplitudes (reflecting global inhibition received by the recorded PV cell) was on average 0.45 ± 0.11 (*n* = 7; S3 Fig).

These results indicate that, overall, unitary autaptic self-inhibition contributes to a large fraction (approximately 40%) of the global inhibition (mostly perisomatic) that PV cells receive.

### γ-Oscillations induced in layers II/III are efficiently propagated to layer V PV cells

PV cells play a key role in driving network oscillations in the β-γ-frequency range (20–100 Hz) [5,7–10], believed to underlie several cognitive functions, such as attention and sensory representation [5,23]. Importantly, PV-PV synaptic and electrical coupling is important for synchronizing these interneurons during γ-oscillations [7,8,26], and previous evidence indicated that autaptic self-inhibition of PV cells is instrumental for their spike precision in the γ-frequency range [16]. We therefore tested whether autapses, which constitute the major GABAergic output of these interneurons, could modulate PV-cell spike output induced by γ-like activity.

**Fig 5:**
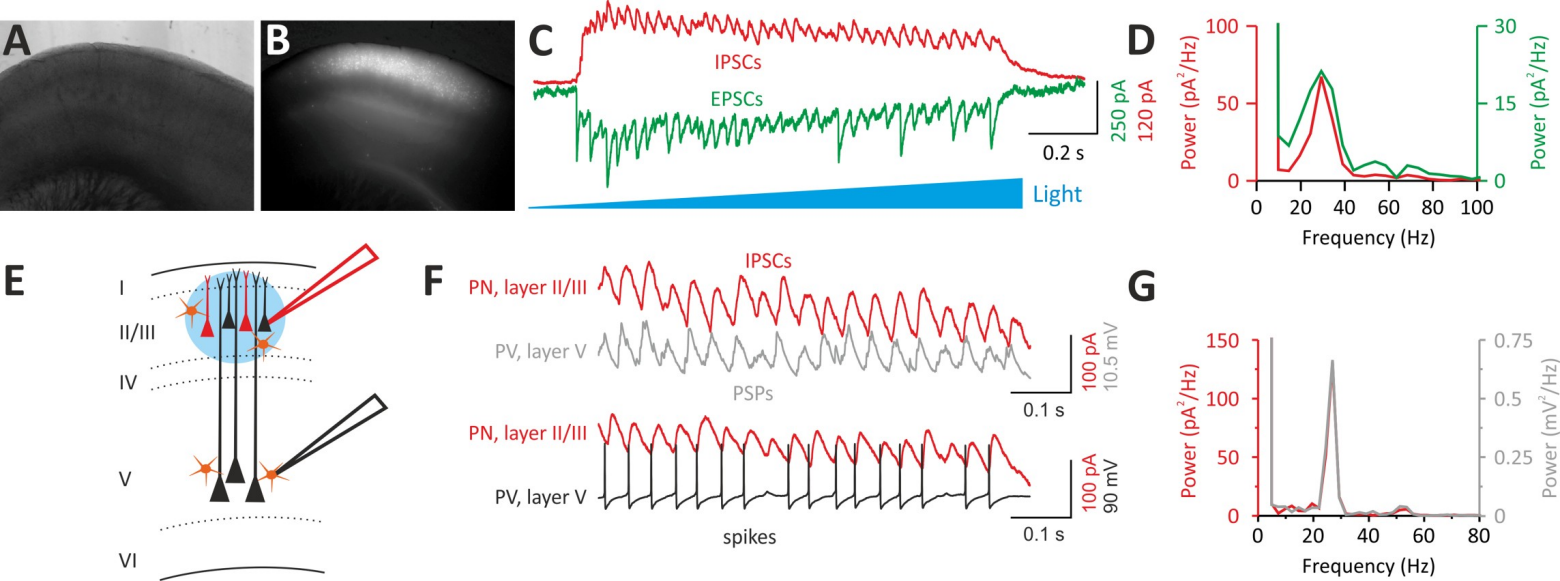
Optogenetically induced γ-oscillations in layer II/III efficiently propagate to layer V PV interneurons. (A, B) Bright-field (A) and fluorescent (B) photomicrographs of an acute cortical slice of a mouse that was electroporated in utero at E15.5 with two plasmids expressing ChR2 and mRFP, respectively. Note the wide expression of mRFP in layer II/III in the barrel field (B). (C) Representative voltage-clamp traces of IPSCs (red) and EPSCs (green) recorded from a ChR2-negative PN of layer II/III in response to a ramp of blue light delivered with an LED coupled to the epifluorescence path of the microscope. IPSCs and EPSCs were isolated by holding the recorded neuron at the reversal potential of glutamate- and GABA-mediated responses, respectively. (D) Power spectra of the IPSCs (red) and EPSCs (green) of the cell of (C). Note the sharp peak at approximately 30 Hz in the γ-frequency range. (E) Scheme of the experimental configuration: a dual patch-clamp recording is established. A ChR2-negative PN in layer II/III is recorded in voltage clamp, and a PV cell in layer V is simultaneously recorded in current clamp: a ramp of blue light is then delivered on layer II/III PN cell bodies. (F) A ramp of blue light induces rhythmic IPSCs in the layer II/III PNs and subthreshold PSPs in the PV cell recorded at its resting potential in layer V. When the PV cell was slightly depolarized, optogenetic activation of ChR2-positive layer II/III PNs induced sustained firing of the PV cell in layer V. (G) The power spectra of IPSCs (red) and PSPs (gray) of the layer II/III PN and layer V PV cell shown in (F) coincide, indicating a good transmission of layer II/III γ-activity across the two cortical layers. ChR2, channelrhodopsin2; E, embryonic day; EPSC, excitatory postsynaptic current; IPSC, inhibitory postsynaptic current; LED, light-emitting diode; mRFP, monomeric red fluorescent protein; PN, pyramidal neuron; PSP, postsynaptic potential; PV, parvalbumin.

We expressed the light-sensitive opsin channelrhodopsin2 (ChR2) in a fraction of layer II/III PNs via in utero electroporation of mouse embryos (Fig 5A and 5B; see Materials and methods). ChR2-negative PNs in the same layer II/III area were recorded where the opsin was expressed. In agreement with previous reports [27–29], illumination of cortical slices with a ramp of blue light induced strong rhythmic activity of both IPSCs and excitatory postsynaptic currents (EPSCs) at approximately 30 Hz (Fig 5C and 5D). Layer II/III PNs project monosynaptically to layer V neurons [27]. We therefore simultaneously recorded layer V PV cells and ChR2-negative PNs in layer II/III (Fig 5E) to measure rhythmic IPSCs in layer II/III PNs and voltage fluctuations of layer V PV cells (see Materials and methods). We found that light-evoked γ-activity in layer II/III was reliably transmitted to layer V PV cells, as shown by subthreshold postsynaptic potentials (PSPs), which oscillated at the same frequency as IPSCs recorded in layer II/III (Fig 5F and 5G). When the membrane potential was slightly depolarized, light activation of a fraction of layer II/III PNs triggered several APs in layer V PV cells (Fig 5F), strongly resembling PV-cell firing activity recorded in vivo [11]. Importantly, the relative power of oscillations remained constant for relatively long periods (20–40 min; S4A–S4C Fig). Moreover, the dominant frequency remained unchanged over long periods (S4D and S4E Fig). This suggests that network properties were constant during our recordings.

These results indicate that optogenetically induced γ-oscillations in layer II/III are faithfully propagated to layer V PV cells, thus allowing studying the role of autaptic self-innervation of these cells during cortical network activity.

### Autaptic neurotransmission facilitates the tuning of PV-cell firing to γ-oscillations

We tested whether the strong inhibitory autaptic conductance occurring after each spike in PV cells is important for synchronizing these interneurons during γ-oscillations. Autaptic responses cannot be measured in physiological low intracellular [Cl^−^], as they overlap with spike AHPs. However, autaptic transmission was shown to modulate AHP duration [30] and ISIs [17] of cortical fast-spiking interneurons.

**Fig 6:**
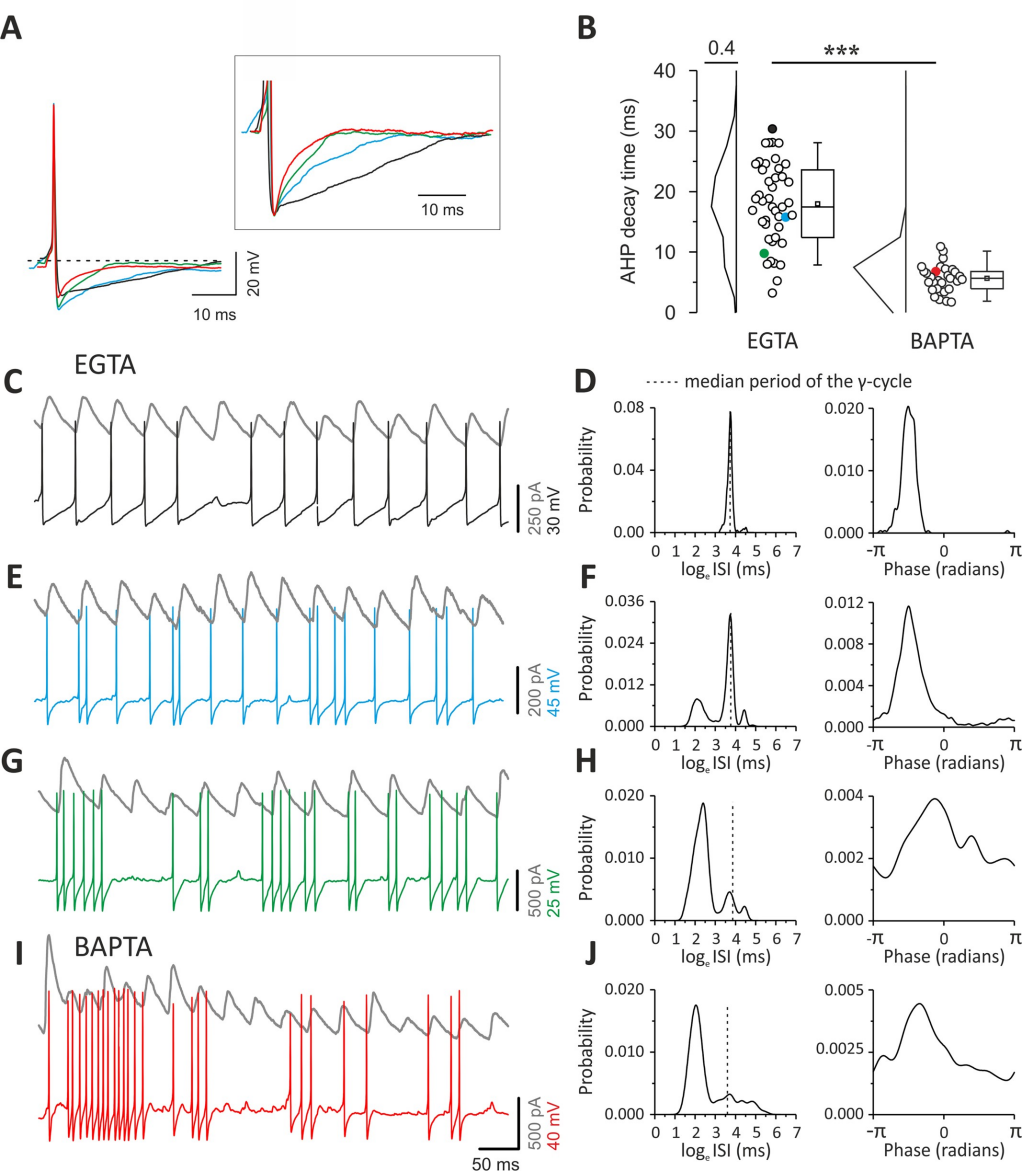
Different AHP durations and firing patterns of PV cells during γ-oscillations. (A) Representative overlapped action potentials (aligned to their peaks) recorded from different PV cells showing different AHP waveform in control (EGTA: black, blue, and green traces) and in the presence of intracellular BAPTA (red trace). Inset: same traces at a larger voltage and time scale, normalized to the negative peak of the AHP. (B) Plots of AHP duration from PV cells recorded with control intracellular solution (EGTA, left) and BAPTA (right). Colors indicate the cells illustrated in (A). (C) Representative traces of oscillating IPSCs recorded from a layer II/III ChR2-negative PN (gray) and a PV cell with slow AHP (same as [A] and [B], black). Note that spikes occur regularly at precise times, relative to the oscillating IPSCs. (D) Distributions of ISIs (left) and phases (right) of the PV cell illustrated in (C) with a black trace. The dotted line indicates the interval corresponding to the median oscillation period. Note the sharp ISI distribution peaking at the oscillation period and the sharp phase distribution. (E, F) Same as (C) and (D) but for the PV cell represented with the blue trace in (A) and (B). Note the appearance of spike doublets (E), yielding multimodal ISI distribution (F, left) and broader phase histogram (right). (G, H) Same as (C–F) but for the cell represented with green trace in (A) and (B). Note the appearance of high-frequency bursts (G) yielding a large peak in the ISI distribution at faster intervals than the oscillation period. Furthermore, note that the phase histogram yielded an even broader profile. (I, J) Same as in (C–H) but for the PV cell intracellularly perfused with BAPTA, illustrated with a red trace in (A) and (B). Note the similar firing behavior of the EGTA cell characterized by the fast AHP and burst firing (green traces in [A, B, G, and H]). Individual numerical data for panel B are provided in Supporting information, S5 Data. AHP, after-hyperpolarization; BAPTA, 1,2-bis(o-aminophenoxy)ethane-N,N,N′,N′-tetraacetic acid; ChR2, channelrhodopsin2; IPSC, inhibitory postsynaptic current; ISI, interspike interval; PN, pyramidal neuron; PV, parvalbumin.

In control conditions (with intracellular 1 mM EGTA), PV cells showed a broad range of AHP durations (Fig 6A and 6B), consistent with varying strengths of autaptic transmission across different PV cells [30]. When autaptic neurotransmission was blocked by intracellular perfusion of BAPTA (Fig 4) [16,20], AHP duration was significantly smaller (17.96 ± 1.02 versus 5.66 ± 0.42 ms; EGTA versus BAPTA, respectively; *p* = 7.75 × 10^−16^; independent *t* test; Fig 6A and 6B) and with a much reduced dispersion between cells (coefficient of dispersion: 2.6 versus 0.9, EGTA versus BAPTA, respectively, *p* = 4.06 × 10^−8^; two-sample test for variance; Fig 6B). This BAPTA-induced shortening of AHP likely resulted from the combined effect of this fast Ca^2+^ chelator on both autaptic transmission and Ca^2+^-activated K^+^ conductances, which are known to shape the AHP waveform [31].

Under control (EGTA) conditions, the specific duration of the AHP determined the coupling of PV-cell spikes with γ-oscillations. Indeed, PV cells with slow AHPs produced spike trains that were regular and strongly coupled to γ-oscillations, as the large majority of APs occurred almost invariably at a precise time during the γ-cycle (Fig 6C). This strong coupling of PV-cell spiking activity with γ-oscillations determined a very sharp, unimodal distribution of ISIs peaking at the γ-oscillation period, as well as a sharp phase coupling histogram (Fig 6D). PV cells with faster AHPs exhibited high-frequency doublets (Fig 6E and 6F) and/or bursts of spikes (Fig 6G and 6H). In these cases, ISI distributions were multimodal, exhibiting peaks at shorter intervals than the oscillation period. Moreover, the spike coupling to γ-phase was increasingly less sharply distributed (Fig 6F and 6H).

Multimodality of ISI distributions and broad spike-phase coupling resulted from an increasing number of spikes with very fast intervening ISIs, not effectively matching the period of ongoing γ-rhythm in layer II/III (Fig 6F and 6H). Control PV cells characterized by the shortest AHPs and consequent weak coupling with γ-oscillations (Fig 6G and 6H) exhibited firing patterns that were similar to PV cells, in which autapses were blocked by intracellular perfusion of BAPTA and characterized by fast AHPs. These PV cells intracellularly perfused with BAPTA consistently produced high-frequency bursts of spikes (Fig 6I), yielding ISI distributions with the largest peak at a faster interval than the oscillation period and broad spike-phase coupling distributions (Fig 6J).

**Fig 7:**
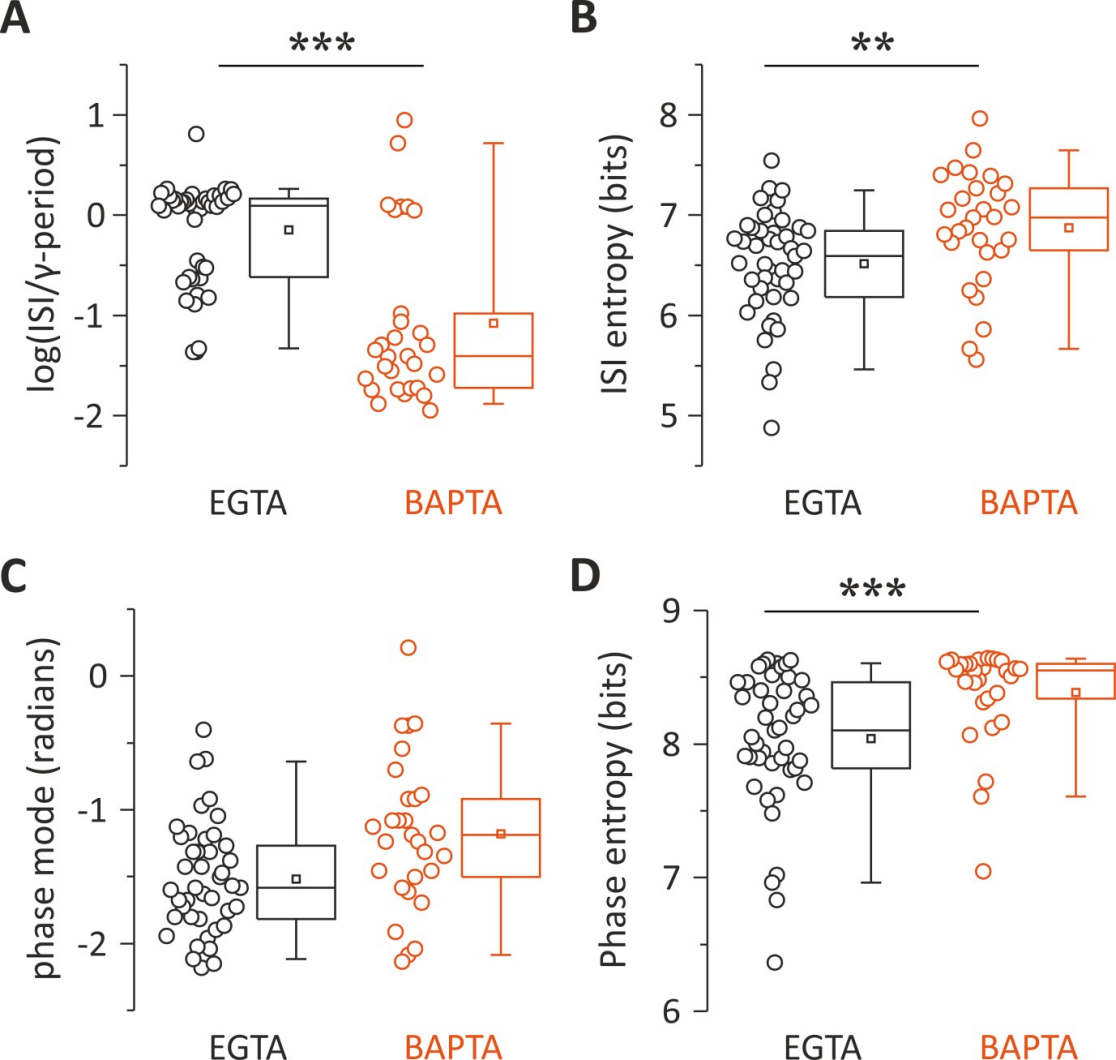
Autaptic neurotransmission facilitates the tuning of PV-cell firing to γ-oscillations. (A, B) Population data and distributions of ISI values relative to γ-period (A) and ISI entropy (B) of PV cells recorded with intracellular EGTA (black) and BAPTA (red). The zero value in the y-axis of the left plot corresponds to the period of the γ-cycles. (C, D) Same as in (A) and (B) but for the peak (mode) of phase distributions as illustrated in Fig 6. **p < 0.01; ***p < 0.001. Individual numerical data for all panels are provided in Supporting information, S6 Data. BAPTA, 1,2-bis(o-aminophenoxy)ethane-N,N,N′,N′-tetraacetic acid; ISI, interspike interval; PV, parvalbumin.

On average, the distribution of ISIs in control (EGTA) cells peaked close to the γ-cycle. Conversely, BAPTA-filled PV interneurons discharged with ISIs not matching the γ-period (mean log ratio: −0.147 ± 0.077 and −1.078 ± 0.155 in EGTA and BAPTA, respectively, *p* = 6.45 × 10^−7^, Wilcoxon rank-sum; Fig 7A). Further, distributions of ISIs were significantly less dispersed in control (EGTA) PV cells as compared with PV cells filled with BAPTA as measured by the log ISI entropy (mean: 6.51 ± 0.08 and 6.87 ± 0.11 bits in EGTA and BAPTA, respectively, *p* = 0.0063, Mann–Whitney; Fig 7B).

The peaks of phase distributions were not significantly different in EGTA and BAPTA (−1.524 ± 0.0613 and −1.189 ± 0.098 radians, respectively; *p* = 0.1, k-test for circular distribution; Fig 7C). However, the dispersion of their distributions were different in control (EGTA) versus BAPTA-filled PV cells, indicating a lesser extent of phase lock induced by intracellular perfusion of BAPTA as measured by the entropy of the phase distribution (mean: 8.041 ± 0.078 and 8.387 ± 0.0689 bits in EGTA and BAPTA, respectively, *p* = 4.6 × 10^−4^; Fig 7D).

AHP modulation by autaptic transmission was shown to occur in the hippocampus [29]. We thus tested whether GABAergic self-inhibition affected AHP in neocortical PV cells. We quantified the actual contribution of autaptic GABAergic transmission to the AHP duration of PV cells. APs were recorded with physiological intracellular chloride and elicited by slow manual depolarization to threshold. We found that gabazine application decreased AHP duration in eight out of 13 cells (61.54%, S5 Fig). This is in agreement with the fraction of PV cells lacking autaptic neurotransmission. Moreover, AHP decay coefficient of variation was significantly decreased upon blocking GABA_A_Rs, consistent with synaptic modulation of spike AHP.

Altogether, these results suggest that the strength of autaptic self-inhibition improves the coupling of PV-cell spiking to γ-oscillations by modulating the duration of their own AHPs.

## Discussion

Here, we found that autaptic transmission is overall the most powerful output from PV cells in neocortical layer V. Autaptic transmission is approximately 3-fold stronger than synaptic inhibition onto PNs and approximately 2-fold larger than PV-PV connections. Moreover, we found that PV cells with strong autaptic transmission produce a weaker synaptic output onto other PV cells and vice versa, thus defining a novel architecture of relative connectivity strength. Despite the existence of a minority of PV cells with stronger PV-PV synaptic than autaptic transmission, self-inhibitory autapses, originating from a single axon, contribute up to approximately 40% of global inhibition (mostly perisomatic) onto PV cells. Strong, reliable, and fast autaptic self-inhibition of PV cells contributes to duration of the AHP and therefore the ISIs of these interneurons, affecting their degree of synchronization with γ-oscillations.

The observation of larger autaptic currents than inhibitory synaptic responses elicited by the same PV cells onto PNs was not due to differences in the number of release sites but to a larger autaptic quantal size. This was confirmed by experiments analyzing asynchronous quantal events at both PV-cell autaptic and PV-PN synaptic sites. A larger quantal size can be ascribed to several causes, including, for example, different subunit composition of GABA_A_Rs, their expression level at postsynaptic sites, their phosphorylation state, and the specific interactions with distinct scaffolding, anchoring, and transsynaptic proteins [32].

Another reason for a smaller quantal size in PNs could be a more distal location of PV-PN synapses as opposed to PV-cell autapses. This could result in more low-pass filtering of synaptic responses with a consequent reduction in their size. Although we cannot exclude that this is the case, we argue against this possibility because PV cells are known to be perisomatic-targeting cells [33]. Indeed, the cell body of large layer V PNs is almost completely innervated by PV-positive inhibitory terminals [34]. This is consistent with the very fast rise time of PV-PN unitary synaptic responses (<1 ms). Finally, although a different quantal size between two synaptic connections is traditionally ascribed to postsynaptic factors, we cannot exclude that the difference in q could be due to a different amount of neurotransmitter released by each vesicle at each individual synapse. Future studies will be necessary to pinpoint the molecular mechanism underlying the difference in quantal size between autapses onto PV cells and synapses onto PNs.

Curiously, connections between PV cells showed a connectivity logic dictated by their actual autaptic strength. Although self-contacts were generally stronger than heterosynaptic connections with other PV cells, autapses were weaker in a minority of cases (approximately 38%). In both introverted and extroverted PV cells, the difference between autaptic and synaptic strength was due to a higher or lower number of release sites, and thus it was due to anatomical specificities. Similar quantal size at autaptic and synaptic connections between PV cells indicates that postsynaptic sensitivity to released GABA at autaptic contacts is equivalent to that of synaptic connections. This could be due to expression of molecularly similar postsynaptic receptor clusters and similar degree of autaptic and synaptic filtering.

The existence of extroverted and introverted PV cells prompts the question of whether they belong to different cell types. Whereas we detected no changes of passive and firing properties of introverted and extroverted, we cannot exclude differential morphology and/or connectivity patterns. Alternatively, the differential strength of self- versus heterosynaptic inhibitory contacts could be due to activity-dependent plasticity of GABAergic connections from PV cells. Indeed, it has been shown that postsynaptic activity could modulate the strength of GABAergic synapses from PV cells in the visual [35] and somatosensory cortex [36]. Future studies, including detailed anatomical analyses of synaptic connections from PV cells to different targets, will be necessary to reveal the mechanisms underlying the differential autaptic and synaptic strength onto PV cells. Importantly, our BQA approach yielded values that were independently confirmed for quantal size (S1 Fig) and, regarding the number of release sites, in good agreement with published anatomical data for PV-PN synapses [37].

Functional autaptic neurotransmission represents a powerful form of fast disinhibition of PV cells. Accumulating evidence indicates that disinhibitory circuits play crucial roles for several cognitive functions [3,4,38,39]. In particular, disinhibition operated by vasoactive intestinal peptide (VIP) cells [13,40] may be crucial for auditory discrimination [38], memory retention in prefrontal cortex [41], and other forms of associative learning and memory [39]. Importantly, however, VIP cell–mediated disinhibition requires multisynaptic circuits, and because it occurs over relatively long (hundreds of ms) time windows, it might be important for modulating the information carried by a whole spike train according to a traditional rate-coding scheme. By contrast, autaptic self-inhibition of PV cells accounts for approximately 40% of the global perisomatic inhibition they received (assessed with two different methods estimating a similar domain of inhibitory synapses impinging on single PV cells). It is fast (occurring at a millisecond timescale) and activated by single spikes. Autaptic disinhibition of PV cells should therefore be crucial for encoding information carried by the precise timing of individual spikes within a high-frequency train. Indeed, we show that fast GABAergic self-inhibition of PV cells modulates the locking of their spike timing to network oscillations in the β-γ-frequency range.

Autaptic transmission occurs immediately after single APs, thus modulating the duration of the AHPs of PV cells during trains of spikes (S5 Fig) [17,30]. In control (EGTA) conditions, we found a broad range of AHP durations. This is consistent with heterogeneous autaptic strengths among several PV cells and lack of functional self-innervation in some cases (approximately 30%). Indeed, gabazine application affected AHP duration in approximately 62% of PV cells recorded in physiological intracellular chloride (S5 Fig). Accordingly, autaptic blockade by intracellular BAPTA invariably produced fast AHPs and high-frequency firing. Relatively long-lasting AHPs correlated with a strong synchronization of PV cells’ output with γ-oscillations. Importantly, the tight locking of PV-cell spikes to γ-activity shown here was similar to that recorded from PV cells in the visual cortex in vivo in the absence and presence of sensory stimuli [11].

Interestingly, faster AHPs enable the generation of high-frequency doublets and/or bursts of spikes. This activity could be detected in virtually all cells intracellularly perfused with BAPTA. The sharpening of PV-cell AHPs induced by intracellular BAPTA was likely due to the blockade of autaptic transmission combined with the impairment of Ca^2+^-activated K^+^ channels, both contributing to AHP peak and duration [31]. In fact, the duration of AHP from PV cells, in which autaptic transmission was blocked by gabazine, was in the same range but not identical to AHPs measured during γ-activity. This could be due to the different sources of AP generation (network versus intracellular current injection). Importantly, a minority of cells recorded with intracellular control (EGTA) conditions exhibited sharp AHPs similar to those recorded with intracellular BAPTA. This is consistent with the fraction of PV interneurons lacking functional autaptic transmission, but with intact Ca^2+^-activated K^+^ channels. The heterogeneity of AHP durations and firing behaviors during γ-activity in control PV cells suggests that the instantaneous spike frequency is highly controlled by autaptic strength. A strong GABAergic conductance, reliably activated with a high release probability immediately after each spike, shapes the window of opportunity to fire a subsequent spike. Therefore, rhythmic glutamatergic activation of PV cells by layer II/III PNs and the strong, fast, and reliable autaptic self-inhibition work in synergy to lock PV-cell firing to the oscillation period. It is known that mutual inhibition between PV cells plays a crucial role during synchronous network activity [9,10]. We therefore speculate that spike timing regulation through autaptic self-inhibition might strongly influence the output spike timing of several PV cells in a millisecond timescale. This could effectively facilitate the synchronization of networks of PV cells during the emergence of fast oscillations.

Overall, our results indicate that self-inhibition of PV cells via autaptic neurotransmission is among the most powerful connections from this cell type within the layer V cortical microcircuit, promoting their spiking synchronization during γ-oscillations. GABAergic autaptic self-inhibition of PV cells could therefore be an important mechanism underlying the key role of these cells during cognitive-relevant network oscillations, with possible crucial consequences in both physiological and pathological cortical operations.

## Materials and methods

### Ethics statement

This work was carried out on mice. Experimental procedures followed national and European (2010/63/EU) guidelines and were approved by the authors’ institutional review boards and national authorities (French Ministry of Research, protocol ID: 02230.02). All efforts were made to minimize suffering and reduce the number of animals. For survival surgery experiments (in utero electroporation), pregnant female mice were anesthetized with 1%–2% isoflurane. For the nonsurvival procedure (preparation of acute cortical slices), mice were deeply anesthetized with isoflurane before decapitation.

### Animals

Experiments were performed on C57BL/6J mice obtained by breeding mice expressing the Cre recombinase under the control of PV promoter (PV-cre; jax line 008069) to a reporter line harboring a loxP-flanked STOP cassette associated to the red fluorescent protein variant tdTomato (line Ai14 jax line 007914). The resulting mouse line (PV-cre::tdTomato) allowed recognition of PV interneurons in acute slices. Indeed, tdTomato-expressing cells had typical multipolar morphology, aspiny dendrites, and fast-spiking behavior (not shown).

### In utero electroporation

E15.5 embryos from timed-pregnant PV-cre female mice bred with tdTomato males were anesthetized with 1%–2% isoflurane. The abdomen was cleaned with 70% ethanol and swabbed with betadine. Buprenorphine (0.05 mg/kg) was administered subcutaneously for preoperative analgesia, and local anesthetic bupivacaine (2.5 mg/kg) was injected between the skin and the abdomen 5 min before incision. A midline ventral laparotomy was performed, and the uterus was gently exposed and moistened with PBS prewarmed at 37°C. pCAG-mRFP (0.8 μg/μl) (Addgene #28311) [42] plasmid DNA was mixed with pCAG-ChR2-Venus (0.8 μg/μl) (Addgene #15753) [43] and Fast Green (0.025%; Sigma) in saline solution (PBS).

Each embryo was injected with the mix DNA solution through the uterine wall into the lateral ventricle using pressure-controlled beveled glass capillaries (WPI micropipette beveler). After each injection, tweezer disk electrodes (platinium 5 mm round, Sonidel) were positioned at a 0° angle with respect to the rostral–caudal axis of the head of each embryos, and voltage pulses (5 pulses, 40 V; 50 ms; 5 Hz) were applied to electroporate the DNA (square wave electroporator, Nepa Gene). The uterine horn containing the embryos was then placed back into the peritoneal cavity and moistened with PBS. The abdomen and skin were sutured, and the latter was cleaned with betadine. The procedure typically lasted a maximum of 40 min starting from anesthesia induction. Pups were born by natural birth and screened for location and strength of transfection by transcranial epifluorescence under a fluorescence stereoscope.

### In vitro slice preparation

Naïve or in utero electroporated mice were deeply anesthetized with isoflurane and decapitated, and the brains were quickly removed. Coronal slices were prepared from somatosensory cortex of mice (aged P15–P25) using a vibratome (Leica VT1200 S) in a free or reduced sodium cutting solution (4°C). Slices were initially stored at 34°C for 30 min in standard or reduced sodium solution (ASCF) and then at room temperature for at least 1 h before being transferred to a submerged recording chamber maintained at approximately 30°C.

For unitary autIPSC and synIPSC experiments, coronal slices (350 μm thick) were obtained from somatosensory cortex using a free sodium cutting solution containing (in mM) choline (118), glucose (16), NaHCO_3_ (26), KCl (2.5), NaH_2_PO_4_ (1.25), MgSO_4_ (7), CaCl_2_ (0.5),_,_ pyruvic acid (3), myo-inositol (3), and ascorbic acid (0.4) gassed with 95% O_2_ and 5% CO_2_. Then, slices were stored in oxygenated standard ASCF (in mM): NaCl (126), KCl (2.5), CaCl_2_ (2), MgSO_4_ (1), NaH_2_PO_4_ (1.25), NaHCO_3_ (26), and glucose (20) (pH 7.4).

For photoinduced γ-oscillation experiments, 400-μm-thick coronal somatosensory cortical slices were prepared from the transfected hemisphere. Slices were cut and stored in oxygenated reduced sodium ACSF containing (in mM) NaCl (83), sucrose (72), glucose (22), NaHCO_3_ (26), KCl (2.5), NaH_2_PO_4_ (1), MgSO_4_ (3.3), CaCl_2_ (0.5), pyruvic acid (3), myo-inositol (3), and ascorbic acid (0.4) (pH 7.4).

### Electrophysiology

#### Unitary autIPSCs and synIPSCs

Recordings were obtained in standard ACSF at 30°C from PV-PV cells pairs and PV-PN pairs of layer V primary barrel somatosensory cortex. Neuron types were visually determined using infrared video microscopy. PV interneurons were visible as tdTomato-positive fluorescent cells, whereas PNs were identified by their large soma and emerging apical dendrite together with firing behavior. Whole-cell voltage-clamp recordings were obtained with patch pipettes (2–4 MΩ) filled with a high [Cl^−^] intracellular solution containing (in mM) K-gluconate (70), KCl (70), HEPES (10), EGTA (1), MgCl_2_ (2), MgATP (4), and NaGTP (0.3) or K-gluconate (35), KCl (70), HEPES (10), 4K-BAPTA (20), CaCl_2_ (2), MgATP (4), and NaGTP (0.3); pH 7.2 adjusted with KOH; 290 mOsm; for EGTA and BAPTA experiments, respectively. GABA_A_R-mediated IPSCs were isolated by adding DNQX (10 μM) in the bath perfusion and recorded at a holding potential of −80 mV or −70 mV. For mIPSCs recordings, DNQX and TTX (1 μM) were added to the bath perfusion. When indicated, SR95531 (6-imino-3-[4-methoxyphenyl]-1[6H]-pyridazine-butanoic acid hydribromide) (gabazine, 10 μM) was also applied by bath perfusion to block GABA_A_Rs. All drugs were obtained from Tocris Cookson (Bristol, United Kingdom).

#### Photoinduced γ-oscillations

After being transferred to the submerged recording chamber, slices were superfused with modified ACSF containing (in mM) NaCl (119), KCl (2.5), CaCl_2_ (2.5), MgSO_4_ (1.3), NaH_2_PO_4_ (1.3), NaHCO_3_ (26), and glucose (20) (pH 7.4) maintained at 30°C. Before starting recordings, slices were carefully examined to check mRFP expression in layer II/III of the somatosensory cortex. Whole-cell, voltage-clamp recordings of photoinduced γ-oscillations were obtained from ChR2-negative PNs identified by the pyramidal shape of their soma, the emerging apical dendrite, and the absence of mRFP and tdTomato fluorescence. Patch pipettes were filled with a cesium-based low [Cl^−^] intracellular solution containing (in mM) CsMeSO4^−^ (125), CsCl (3), HEPES (10), EGTA (5), MgCl_2_ (2); MgATP (4), NaGTP (0.3), QX314-Cl (5); pH 7.2 corrected with CsOH; 290 mOsm. IPSCs and/or EPSCs were recorded at GluR and GABA_A_R reversal potentials, respectively. Simultaneous current-clamp recordings were obtained from layer V tdTomato-positive fluorescent PV cells located within the same cortical column of the layer II/III PN using a K-gluconate-based low [Cl^−^] intracellular solution containing (in mM) K-gluconate (120), KCl (13), HEPES (10), EGTA (1), MgCl_2_ (2), MgATP (4), and NaGTP (0.3) or K-gluconate (103), KCl (13), HEPES (10), 4K-BAPTA (20), CaCl_2_ (2), MgATP (4), NaGTP (0.3); pH 7.2 adjusted with KOH; 290 mOsm; for EGTA and BAPTA conditions, respectively. This intracellular Cl^−^ concentration yielded a calculated reversal potential for GABA-mediated responses of −52.3 mV. This is in line with previous studies (in both the neocortex and hippocampus) that used gramicidin perforated patch recordings and estimated this specific E_Cl_ selectively in PV cells [44,45].

### Photo-stimulation

Photo-stimulation was induced using a blue LED (λ = 470 nm, OptoLED Lite, Cairn Research, Faversham, UK) collimated and coupled to the epifluorescence path of the microscope (BX51WI; Olympus). Light was delivered through a 60× (1.0 NA) water immersion lens, centered on layer II/III. The light intensity and waveform were controlled by the analog output of a digitizer (Digidata 1440A, Molecular Devices, San Jose, CA, United States). Light ramps had a duration of 1–3 s and a slope of 0.1–0.8 mW s^−1^, started at zero intensity, and reached a final intensity of 0.3–1.6 mW s^−1^. The slope was adjusted in each slice to obtain a robust rhythmic activity in the gamma frequency range with a stable power for the entire duration of the stimulus. Light ramps were repeated with a 60-s interval.

### Data acquisition and analysis

Signals were amplified using a Multiclamp 700B patch-clamp amplifier (Molecular Devices, USA), sampled at 20 KHz, and filtered at 2 KHz or 10 KHz in voltage-clamp and current-clamp modes, respectively. Voltage measurements were not corrected for liquid junction potential. Access resistance was <20 MΩ and monitored throughout the experiment. Recordings were discarded from analysis if the resistance changed by >20% over the course of the experiment. Data were analyzed using pClamp (Molecular Devices, USA), Origin (OriginLab, Northampton, MA, USA), MATLAB (MathWorks, Natick, MA, USA), and custom written scripts and software.

#### Unitary autIPSCs and synIPSCs

A brief (0.2–0.6 ms) depolarizing current step was injected in the presynaptic PV interneuron from PV-PV or PV-PN pairs. Voltage jumps were calibrated for each stimulated PV cell to a value ranging between –20 and 0 mV from the holding potential to reduce the contribution of K^+^-mediated conductance of the action current, contaminating the autaptic response. GABAergic autaptic and synaptic responses were recorded in the stimulated PV cell itself and in the paired cell (PV or PN), respectively, in whole-cell voltage-clamp mode. For quantal parameter analysis, responses were recorded at two extracellular Ca^2+^ concentrations (1.5 and 2.0 mM) to induce intermediate and high release probabilities, respectively. Gabazine was applied at the end of the recordings to subtract the stimulus waveform and the isolated action current to autaptic responses (Fig 1A and 1C). The IPSCs’ amplitude was estimated as the current from the baseline before the onset of the stimulus to the peak on control or subtracted trace when gabazine was applied. Data were analyzed using pClamp (Molecular Devices, USA), Origin (Microcal, USA), and MATLAB (Mathworks, USA) software.

#### BQA

Quantal parameters were estimated using an improved implementation of BQA [24] as described previously [46,47]. Briefly, synaptic or autaptic currents were measured in the presence of two different Ca^2+^ concentrations (1.5 and 2 mM) corresponding to intermediate and high release probabilities. BQA was performed only for experiments in which at least 50 stable responses per condition could be recorded, followed (in the case of autaptic connections) by application of gabazine in the presence of both Ca^2+^ concentrations in order to subtract the profile of the action currents, which is subject to changes following reduction of Ca^2+^.

In contrast to multiple probability fluctuation analysis (MPFA) [48], which relies on parabolic fits to the variance–mean relationship of synaptic currents, BQA models the distribution of all amplitudes observed at different release probabilities. The advantage this confers on BQA is that quantal parameters can be reliably estimated from the few response measurements obtained from only two different release probabilities [24]. Quantal parameters were estimated as the median value of the posterior distributions. The BQA implementation was modified in the selection of the marginal priors for the number of release sites. Contrary to our previous implementation [24], in which the marginal priors for the probability of release and the uniquantal coefficient of variation were assigned according to Jeffrey’s rule, whereas the number of release sites had a uniform prior, here we applied Jeffrey’s rule to the number of release sites as well, resulting in a reciprocal, rather than uniform, prior distribution.

#### Miniature inhibitory synaptic events

ACSF containing a high concentration of K^+^ (approximately 20 mM) was applied using a pressure system (puff) through a glass pipette located near the cell body of the recorded PV interneuron to depolarize axon terminals impinging onto the recorded neuron. High-K^+^ puffs induced global asynchronous release of GABA that could be detected as a substantial increase in the frequency of mIPSCs. mIPSCs were recorded during successive sequences of baseline activity and throughout puff application (3–6 s, 1 puff/min) for at least 20 min. Miniature GABAergic events (induced by high-K^+^ puffs and asynchronous release) were detected using a custom written software (Detector, courtesy J. R. Huguenard, Stanford University; S1 and S2 Figs). Briefly, individual events were detected on a differentiated copy of the raw current trace with a threshold-triggered process. Detection frames were inspected visually to ensure that the detector was working properly. mIPSC frequency was calculated for successive 1-s time windows. For each puff application, the relative mIPSC frequency was estimated as the ratio between the maximum mean frequency (1-s bin) during puff application and the average of the mean frequencies for the entire baseline duration. To evaluate the percentage of mIPSC frequency decrease, we compared the relative frequency (average of three successive values) at the beginning of the recording (5–10 min after whole-cell configuration establishment) to the relative frequency after the block of autaptic currents by BAPTA (approximately 20 min after whole-cell configuration establishment).

#### Extracellular stimulation

For the experiments detailed in S3 Fig, a monopolar stimulating electrode was placed at approximately 100 μm lateral to the cell body of the recorded PV cell. After determining threshold responses (yielding occasional failures), an input–output curve was generated to determine the maximal synaptic response. We then evoked 10–20 trials of unitary autaptic and extracellularly evoked synaptic responses. All experiments were performed in the continuous presence of DNQX (10 μM), and gabazine (10 μM) was systematically applied at the end of each experiment to subtract the stimulation artifacts.

#### Firing properties of layer V PV cells during photoinduced γ-activity

Bursts of γ-activity were evoked by light stimulation of ChR2-positive PN cell bodies in layer II/III. While recording from a layer V PV interneuron, a simultaneous recording of a layer II/III ChR2-negative PN was used to determine the period of the γ-activity. Rhythmic synaptic events evoked by light stimulation were detected using Detector software as described above for mIPSCs. Spikes of layer V PV cells were extracted using a threshold of −10 mV on the membrane potential trace. PSC cycles were measured, and the timing of each spike in the PV neuron was expressed as a phase relative to the peak of each γ-oscillation. ISIs and phase distributions were computed for each cell using custom written scripts (MATLAB). Variability of firing was evaluated using the entropy of the log interval distribution [49,50], whereas the dispersion of pericycle spike times was quantified using the entropy of the corresponding phase distribution [49].

The circular statistic toolbox of MATLAB was used to compute parameters of phase distributions and their associated statistical tests, as indicated in the text.

AHP duration of single APs was measured as the 10%–90% decay time setting the baseline right before the spike (5-ms window). Hence, isolated spikes were selected for this analysis because the decay time of AP being part of doublets or burst could be contaminated by the generation of the following one. The average value of the AHP decay time of 10–20 spikes was considered for each PV cell.

#### Statistics

Because most data distributions were not normal, unless indicated in the text, we used nonparametric significance test, Wilcoxon’s signed-rank test, and Wilcoxon’s rank-sum test for paired and unpaired data, respectively.

## Supporting information

S1 Fig Quantal size estimated from asynchronous release at PV-cell autaptic and PV-PN synaptic sites. (A) Representative voltage-clamp traces from a PV-PN pair showing robust autaptic and synaptic asynchronous release at both sites in response to a high-frequency spike train elicited in the PV cell (200 Hz, 1 s). The bottom panel illustrates the asynchronous autaptic and synaptic release at a faster time scale. The gray box indicates the time window (400–500 ms) used for the analysis. (B) Amplitude distribution of asynchronous autaptic (top) and synaptic (bottom) events of the pair of (A). (C) Population plot illustrating the median asynchronous IPSC amplitude obtained from paired (filled circles) and unpaired (open circles) recordings. *p < 0.05 paired sample Wilcoxon signed-rank test (gray bar); **p < 0.01 Mann–Whitney test for all data. Individual numerical data for panel C are provided in Supporting information, S7 Data. IPSC, inhibitory postsynaptic current; PN, pyramidal neuron; PV, parvalbumin.
(TIF)

S2 Fig Detection of global inhibition onto PV cells induced by ambient depolarization by high extracellular K^+^. Global inhibition onto single PV cells was estimated as the increase of mIPSC frequency evoked by a local puff of 20 mM KCl, triggering massive Ca^2+^-dependent release of GABA onto the recorded neuron (Fig 4). Shown is a snapshot of the mIPSC detection software before (left) and after (right) the high-KCl puff, illustrating the ability of detecting high-frequency synaptic events in response to ambient depolarization. Events were detected based on a threshold-crossing algorithm on the derivative (bottom) of the current traces (top). Vertical lines indicate detected synaptic events. mIPSC, miniature inhibitory postsynaptic current; PV, parvalbumin.
(TIF)

S3 Fig Single-axon autaptic inhibition versus global perisomatic inhibition onto PV cells. (A) Experimental design. In the continuous presence of DNQX, single PV cells were recorded, and a stimulation electrode was placed at approximately 100 μm from the cell body. This allows the activation of both autaptic and synaptic inhibition onto the recorded cell. Single-cell autIPSCs were evoked by brief intracellular depolarizations. (B) Representative voltage-clamp traces of autaptic (left) and extracellularly evoked (right) IPSCs, recorded in the same cell. Gabazine was added at the end of the experiment (purple traces). The subtracted trace (control-gabazine, orange) was used for the analysis. Shown are averages of 10 trials. (C) Population graph illustrating the ratio between unitary autaptic response and global (autaptic + synaptic) responses obtained with extracellular stimulation. Individual numerical data for panel C are provided in Supporting information, S8 Data. autIPSC, autaptic inhibitory postsynaptic current; DNQX, 6,7-dinitroquinoxaline-2,3,dione; IPSC, inhibitory postsynaptic current; PV, parvalbumin.
(TIF)

S4 Fig Stability of light-evoked γ-oscillations. (A) Representative whole-cell IPSC traces obtained from a ChR2-negative PN in layer II/III at the reversal potential for glutamate-mediated responses at three time points; t_0_ is the first trial of the γ-oscillation experiment. (B) Power spectra of the recordings of (A). (C) Average relative power over time (*n* = 21). (D) Frequency of oscillations for all experiments. (E) Relative mean peak frequency over time. Note the stability of the rhythmic activity in these experiments. Individual numerical data for panels C–E are provided in Supporting information, S9 Data. ChR2, channelrhodopsin2; IPSC, inhibitory postsynaptic current; PN, pyramidal neuron.
(TIF)

S5 Fig Autaptic modulation of spike AHPs in PV cells. (A) Representative current-clamp recordings of two overlapped action potentials recorded in a PV cell before (control, black) and after applying the GABA_A_R antagonist gabazine (gray). The intracellular chloride concentration was identical to that used in PV cells for γ-oscillation experiments. Spikes were evoked by gradual depolarization until reaching firing threshold (−45 mV). (B) Population graph illustrating gabazine effect on AHP 90%–10% decay time. (C) Population graph showing decay time CV before and after gabazine application. *p < 0.05 paired sample Wilcoxon signed-rank test. Individual numerical data for panels B and C are provided in Supporting information, S10 Data. AHP, after-hyperpolarization; CV, coefficient of variation; GABA_A_R, GABA_A_ receptor; PV, parvalbumin.
(TIF)

S1 Data Individual numerical data for panels B, C, E, and F of Fig 1.
(XLSX)

S2 Data Individual numerical data for panels B, C, F, G, H, and I of Fig 2.
(XLSX)

S3 Data Individual numerical data for panels C–H of Fig 3.
(XLSX)

S4 Data Individual numerical data for panels C and I of Fig 4.
(XLSX)

S5 Data Individual numerical data for panel B of Fig 6.
(XLSX)

S6 Data Individual numerical data for all panels of Fig 7.
(XLSX)

S7 Data Individual numerical data for panel C of S1 Fig.
(XLSX)

S8 Data Individual numerical data for panel C of S3 Fig.
(XLSX)

S9 Data Individual numerical data for panels C–E of S4 Fig.
(XLSX)

S10 Data Individual numerical data for panels B and C of S5 Fig.
(XLSX)

## Abbreviations

ACSF: artificial cerebrospinal fluid
AHP: after-hyperpolarization
AP: action potential
autIPSC: autaptic inhibitory postsynaptic current
BAPTA: 1,2-bis(o-aminophenoxy)ethane-N,N,N′,N′-tetraacetic acid
BQA: Bayesian quantal analysis
ChR2: channelrhodopsin2
DNQX: 6,7-dinitroquinoxaline-2,3,dione
EPSC: excitatory postsynaptic current
GABA_A_R: GABA_A_ receptor
IPSC: inhibitory postsynaptic current
ISI: interspike interval
mIPSC: miniature inhibitory postsynaptic current
PN: pyramidal neuron
PSP: postsynaptic potential
PV: parvalbumin
synIPSC: synaptic inhibitory postsynaptic current
TTX: tetrodotoxin
VIP: vasoactive intestinal peptide
