# Supplementary figures and images for: Strong preference for autaptic self-connectivity of neocortical PV interneurons facilitates their tuning to γ-oscillations
Source: PLoS Biol. 2019 Sep 4;17(9):e3000419. doi: 10.1371/journal.pbio.3000419 (PMC6726197; doi:10.1371/journal.pbio.3000419)

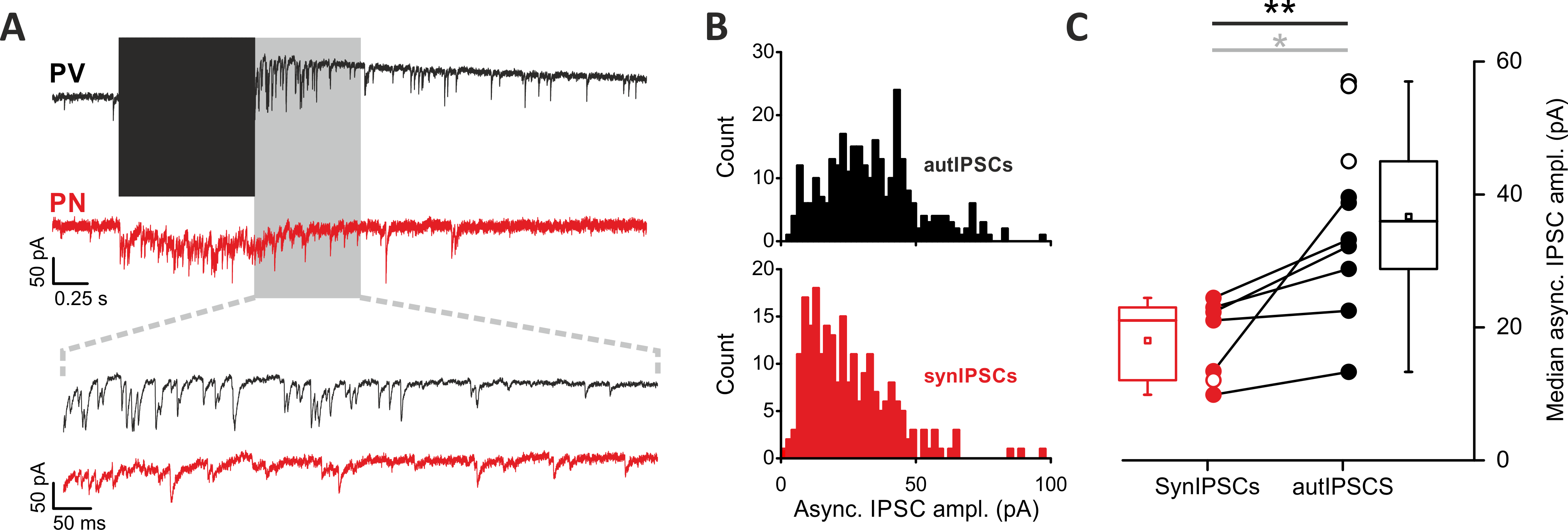

Supplement: S1 Fig — (A) Representative voltage-clamp traces from a PV-PN pair showing robust autaptic and synaptic asynchronous release at both sites in response to a high-frequency spike train elicited in the PV cell (200 Hz, 1 s). The bottom panel illustrates the asynchronous autaptic and synaptic release at a faster time scale. The gray box indicates the time window (400–500 ms) used for the analysis. (B) Amplitude distribution of asynchronous autaptic (top) and synaptic (bottom) events of the pair of (A). (C) Population plot illustrating the median asynchronous IPSC amplitude obtained from paired (filled circles) and unpaired (open circles) recordings. *p < 0.05 paired sample Wilcoxon signed-rank test (gray bar); **p < 0.01 Mann–Whitney test for all data. Individual numerical data for panel C are provided in Supporting information, S7 Data. IPSC, inhibitory postsynaptic current; PN, pyramidal neuron; PV, parvalbumin. (TIF) [file pbio.3000419.s001.tif]

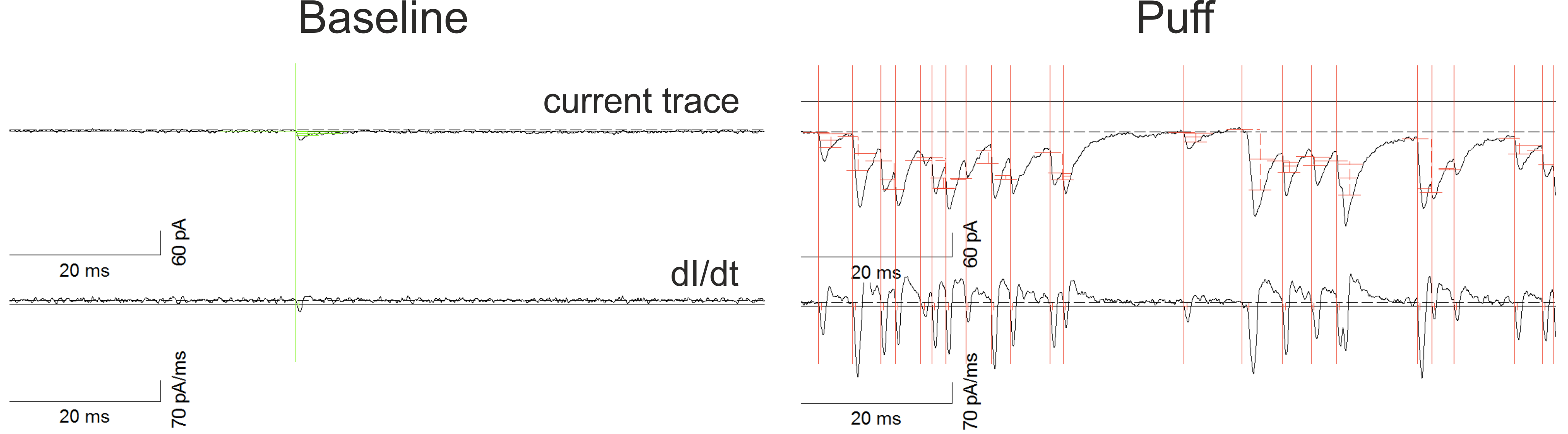

Supplement: S2 Fig — Global inhibition onto single PV cells was estimated as the increase of mIPSC frequency evoked by a local puff of 20 mM KCl, triggering massive Ca2+-dependent release of GABA onto the recorded neuron (Fig 4). Shown is a snapshot of the mIPSC detection software before (left) and after (right) the high-KCl puff, illustrating the ability of detecting high-frequency synaptic events in response to ambient depolarization. Events were detected based on a threshold-crossing algorithm on the derivative (bottom) of the current traces (top). Vertical lines indicate detected synaptic events. mIPSC, miniature inhibitory postsynaptic current; PV, parvalbumin. (TIF) [file pbio.3000419.s002.tif]

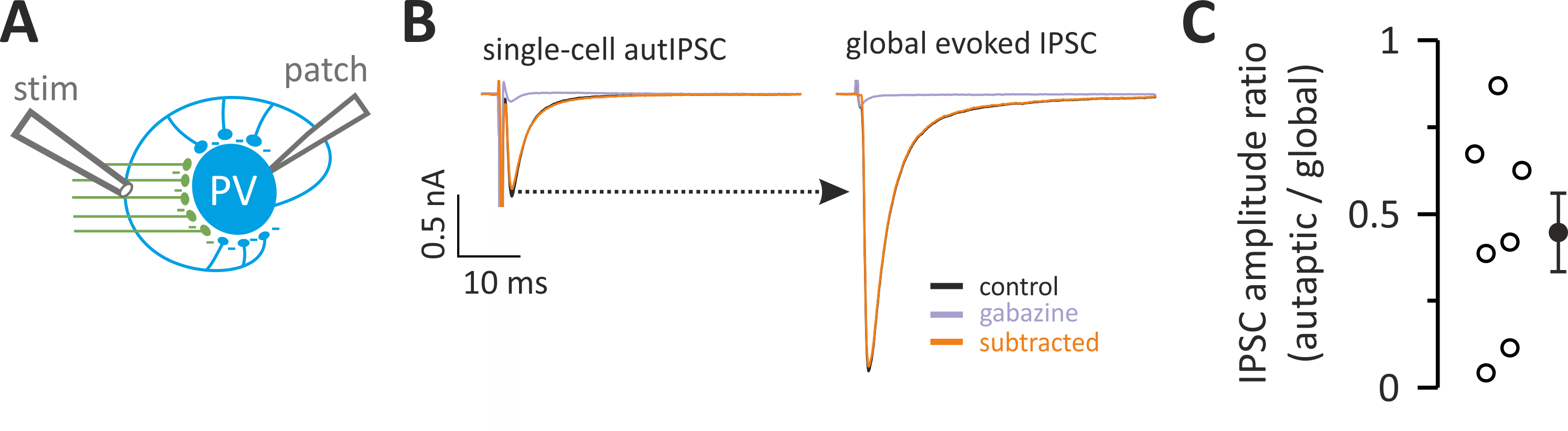

Supplement: S3 Fig — (A) Experimental design. In the continuous presence of DNQX, single PV cells were recorded, and a stimulation electrode was placed at approximately 100 μm from the cell body. This allows the activation of both autaptic and synaptic inhibition onto the recorded cell. Single-cell autIPSCs were evoked by brief intracellular depolarizations. (B) Representative voltage-clamp traces of autaptic (left) and extracellularly evoked (right) IPSCs, recorded in the same cell. Gabazine was added at the end of the experiment (purple traces). The subtracted trace (control-gabazine, orange) was used for the analysis. Shown are averages of 10 trials. (C) Population graph illustrating the ratio between unitary autaptic response and global (autaptic + synaptic) responses obtained with extracellular stimulation. Individual numerical data for panel C are provided in Supporting information, S8 Data. autIPSC, autaptic inhibitory postsynaptic current; DNQX, 6,7-dinitroquinoxaline-2,3,dione; IPSC, inhibitory postsynaptic current; PV, parvalbumin. (TIF) [file pbio.3000419.s003.tif]

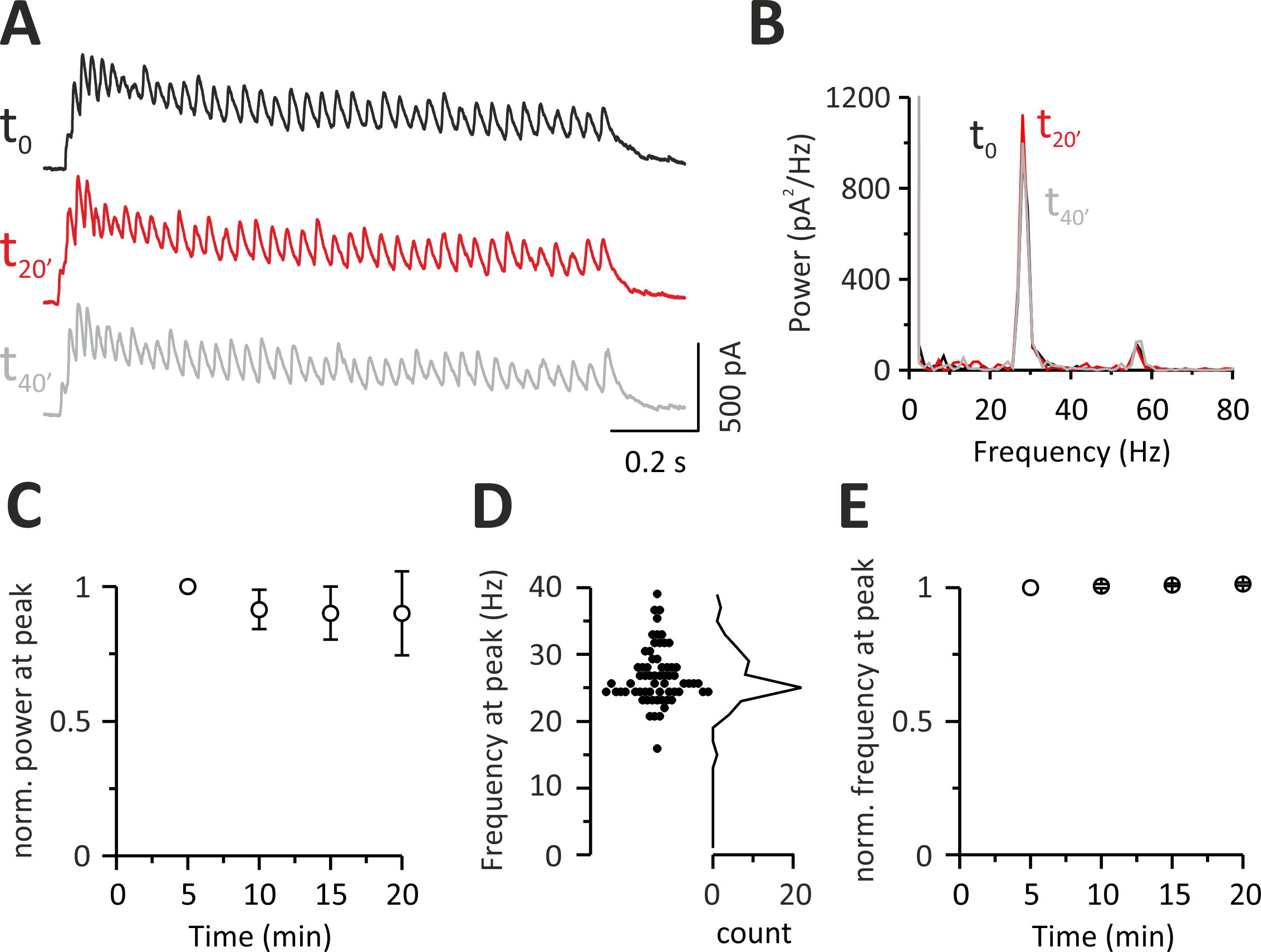

Supplement: S4 Fig — (A) Representative whole-cell IPSC traces obtained from a ChR2-negative PN in layer II/III at the reversal potential for glutamate-mediated responses at three time points; t0 is the first trial of the γ-oscillation experiment. (B) Power spectra of the recordings of (A). (C) Average relative power over time (n = 21). (D) Frequency of oscillations for all experiments. (E) Relative mean peak frequency over time. Note the stability of the rhythmic activity in these experiments. Individual numerical data for panels C–E are provided in Supporting information, S9 Data. ChR2, channelrhodopsin2; IPSC, inhibitory postsynaptic current; PN, pyramidal neuron. (TIF) [file pbio.3000419.s004.tif]

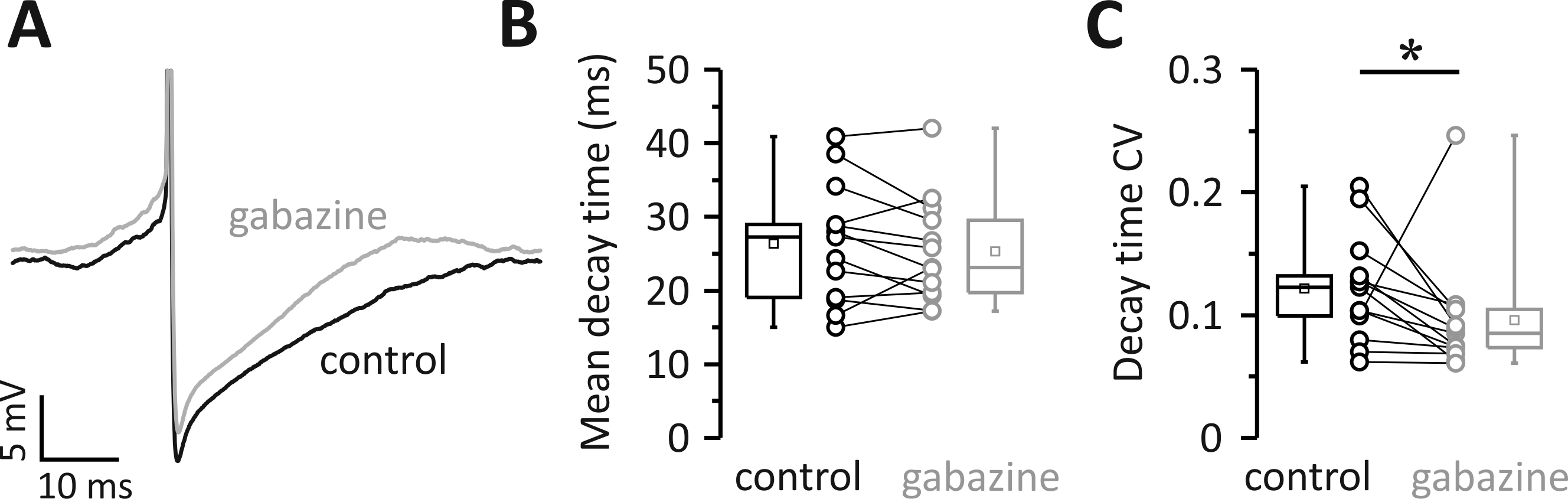

Supplement: S5 Fig — (A) Representative current-clamp recordings of two overlapped action potentials recorded in a PV cell before (control, black) and after applying the GABAAR antagonist gabazine (gray). The intracellular chloride concentration was identical to that used in PV cells for γ-oscillation experiments. Spikes were evoked by gradual depolarization until reaching firing threshold (−45 mV). (B) Population graph illustrating gabazine effect on AHP 90%–10% decay time. (C) Population graph showing decay time CV before and after gabazine application. *p < 0.05 paired sample Wilcoxon signed-rank test. Individual numerical data for panels B and C are provided in Supporting information, S10 Data. AHP, after-hyperpolarization; CV, coefficient of variation; GABAAR, GABAA receptor; PV, parvalbumin. (TIF) [file pbio.3000419.s005.tif]
